# *Candida albicans* colonization in the human colon correlates with a reduction in acetate- and butyrate-producing bacteria, as simulated using the M-SHIME® model

**DOI:** 10.1038/s41522-025-00803-w

**Published:** 2025-08-26

**Authors:** Benoît Marsaux, Warre d’Hoker, Frédéric Moens, Dries Van Elst, Yorick Minnebo, Massimo Marzorati, Tom Van de Wiele

**Affiliations:** 1 ProDigest B.V., Ghent, Belgium; 2 CMET, Ghent University, Ghent, Belgium

**Keywords:** Microbiome, Microbial ecology, Metagenomics, Pathogens

## Abstract

*Candida albicans* is a common gut commensal, typically restricted by the resident microbiota. However, microbiome disruption can enable its outgrowth, increasing the risk of life-threatening candidiasis. Restoring key protective microbes offer a therapeutic strategy, though their identification remains challenging. Using the M-SHIME® model simulating the human proximal colon, we investigated *C. albicans*-bacteriome interactions under eubiotic and dysbiotic conditions. We assessed how clindamycin, ciprofloxacin, and metronidazole modulate *C. albicans* colonization and evaluated associated microbial and metabolic shifts. The effects were antibiotic- and donor-specific: clindamycin facilitated colonization, ciprofloxacin had no impact, and metronidazole showed variable outcomes. Engraftment did not correlate with total bacterial concentration or α-diversity, but with the loss of specific taxa, notably *Lachnospiraceae* and *Bifidobacterium*. These correlations were supported functionally by reductions in acetate and butyrate, suggesting a metabolic mechanism of fungal suppression. This study highlights the role of dysbiosis in *C. albicans* outgrowth and supports targeted microbiome restoration strategies.

## Introduction

The gastrointestinal tract harbors thousands of microbial strains, composed of bacteria, archaea, eukaryotic microbes, and viruses, that are collectively termed the “gut microbiome”^1^. A normal gut microbial ecosystem is highly diverse and prevents outgrowth of opportunistic pathogens through a phenomenon called “colonization resistance”^2^. This colonization resistance involves microbe-microbe interactions, such as competition for nutrients, niches, and binding sites, as well as the release of antimicrobial agents^3–9^. Importantly, environmental stressors, with antibiotics playing a leading role, can disrupt the microbiome, resulting in a condition termed microbial dysbiosis, which is characterized by a weakened colonization resistance^10^, resulting in the emergence of opportunists^11^ such as *Candida albicans*.

*Candida albicans* is a diploid polymorphic fungus that primarily resides in the gut microbiome of warm-blooded animals^12^, including 40 to 80% of healthy individuals in Western countries^13–15^. Normally limited in abundance, antibiotic disruption of the microbiome can result in its increase in humans^16^. Combined with a weakened host immune system, this elevation in *C. albicans* levels poses a risk of life-threatening infections, called candidiasis^17^. Therefore, *C. albicans* is regarded as a pathobiont: a harmless commensal to healthy individuals, but an opportunistic pathogen in vulnerable patients^17,18^. Notably, invasive candidiasis carries a high mortality rate^12^, in part due to the increasing number of isolates that are resistant to common antifungal agents^19^. There is, therefore, a pressing need to find alternative therapeutic approaches.

While it is clear that the gut microbiome plays a strategic role in preventing the transition from a commensal to a pathogenic *C. albicans* phenotype^12^, it is unclear which microbes are involved in this protective effect and what the underlying mechanisms are. Due to their multi-parametric setup, in vitro models that mimic the human digestive tract enable a mechanistic study of the interaction between simulated human colon microbiota and *C. albicans*. Recently, we have demonstrated that the Mucosal Simulator of the Human Intestinal Microbial Ecosystem® (M-SHIME®) model can be used to investigate the interactions between opportunistic fungal pathogens^20^, specifically *Candida*^21^ species, and the human intestinal bacteriome, under conditions of eubiosis (commensal lifestyle) and dysbiosis (pathogenic lifestyle).

In the present study, we used the M-SHIME® model simulating the human proximal colon to investigate the putative associations that exist between *C. albicans* engraftment and bacteriome composition under imposed conditions of dysbiosis. We compared the impact of three antibiotics, namely clindamycin, ciprofloxacin, and metronidazole—all of which are associated with elevated risks to develop candidiasis^22–24^—and evaluated the concomitant shifts in microbial activity and composition. We found that microbiome disturbances occurred in an antibiotic-dependent manner which had differential effects on the colonization ability of *C. albicans*. Overall, our study suggests that the disruption of certain bacterial taxa and function is correlated to *C. albicans* outgrowth.

## Results

### *Candida albicans* grows in a microbiome-free environment under physiological conditions simulating the human proximal colon

**Fig. 1:**
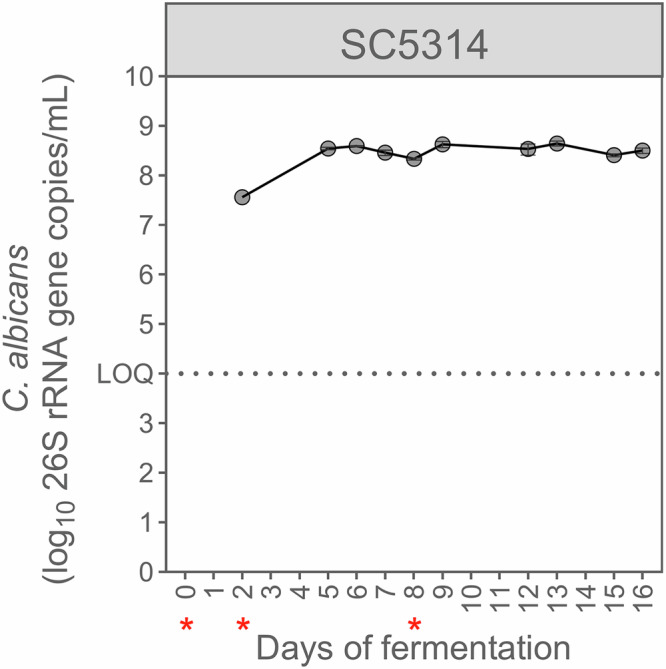
*Candida albicans* persists in the microbiome-free environment of the M-SHIME®, under simulated proximal colon physiological conditions. Concentrations were measured by qPCR (*n* = 3), and data are represented as mean ± standard deviation. *Candida albicans* strain SC5314 was inoculated at days 0, 2, and 8, in a sterile bioreactor, as indicated by red asterisks. The limit of quantification ( = LOQ) is indicated with a dotted line.

We first evaluated the capacity of *C. albicans* to grow under simulated proximal colon conditions in the absence of a microbiome background (see Supplementary Information file for full *C. albicans* concentration time-course). The concentration of *C. albicans*, as quantified by qPCR, was stable from day 5 onwards, reaching ca. 8.5 log_10_(26S rRNA gene copies/mL) (Fig. 1). In addition, its estimated specific growth rate, although varying with time, showed consistent growth throughout the experiment, with a maximal value of ca. 0.08 h^−1^ (Fig. S1). In view of metabolic activity, *C. albicans* produced high ethanol (24.5 ± 3.1 mM) and limited acetate (1.5 ± 0.5 mM) concentrations (Fig. S2). Finally, inoculating the bioreactor a second time at day 8 had no significant impact on *C. albicans* concentration (*p* = 0.17 using two-tailed Welch’s *t*-test) or metabolic production (e.g., *p* = 0.3 for ethanol), indicating that *C. albicans* had reached the carrying capacity in the simulated colon reactor. An additional inoculation was therefore not considered for the subsequent experiments.

### *Candida albicans* only grows in some antibiotic-impaired faecal-derived microbiome environments

**Fig. 2:**
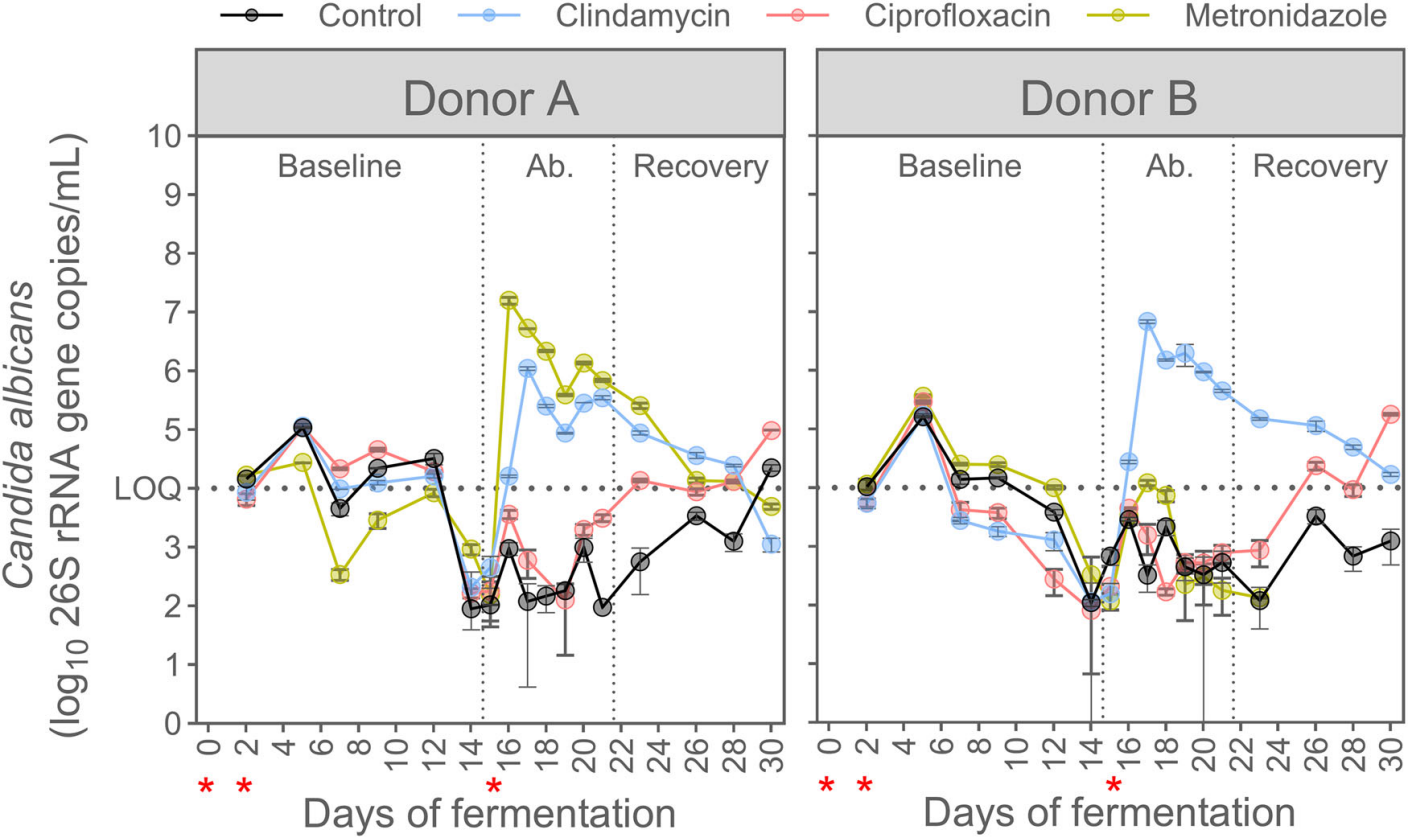
Antibiotic- and donor-dependent persistence of *C. albicans* under simulated proximal colon physiological conditions. The concentrations were measured by qPCR (*n* = 3) in presence of the faecal-derived microbiome from donor A (left) or B (right), and distinct antibiotic treatments. Data are represented as mean ± standard deviation. Samples were collected during the baseline period (days 0 to 14) during which *C. albicans* strain SC5314 was inoculated at days 0 and 2, and throughout the antibiotic treatment (days 14 to 21; Ab. = antibiotic), which started with the re-inoculation of *C. albicans* on day 15, and during the recovery period (days 21 to 30). *Candida albicans* inoculations are indicated by red asterisks. The impact of three antibiotics was compared to a control condition: clindamycin, ciprofloxacin, and metronidazole. The limit of quantification ( = LOQ) is indicated by a horizontal dotted line. The antibiotic treatment period is represented between two vertical dotted lines.

We then assessed to what extent gut microbiota from human origin affected the capacity of *C. albicans* to grow in simulated proximal colon conditions. In the presence of microbiota from donors A and B, *C. albicans* did not engraft, reaching a maximum value of 3 log_10_(26S rRNA gene copy/mL) at day 14 which is below the limit of quantification (Fig. 2). This observation was reproducible across four distinct replicate bioreactors for each donor during the baseline period and was confirmed in the two control conditions (no antibiotic treatment) throughout the remainder of the experiment, in spite of reinoculation at day 15. Calculated *C. albicans* specific growth rate values of 0–0.06 h^−1^ as opposed to the aforementioned 0.08 h^−1^ in the sterile reactor experiment further supported the observed inhibitory effect from the background microbiota towards *C. albicans* (Fig. S1).

We then tested different antibiotics to induce different microbiome impairments and observed *C. albicans* to engraft in an antibiotic and donor-dependent manner (Fig. 2). Clindamycin disruption facilitated *C. albicans* colonization in both donors, while ciprofloxacin did not. In contrast, metronidazole enabled *C. albicans* engraftment in the microbiome derived from donor A but not B. These observations were further confirmed by estimating the specific growth rate, which also indicated that *C. albicans* experienced intermittent periods of cell decay and growth (Fig. S1).

Upon cessation of the clindamycin treatment and subsequent recovery of the microbiota, *C. albicans* was able to persist, albeit at concentrations close to the 4 log_10_(copy/mL) limit of quantification and with limited specific growth rates (0–0.05 h^−1^). In contrast, *C. albicans* did not succeed in persisting in the metronidazole-impaired microbiome from donor A (Fig. 2, Fig. S1). Interestingly, *C. albicans* concentrations increased in ciprofloxacin-impaired microbiomes by the end of the recovery period, exceeding the limit of quantification.

### Total bacteria concentration and overall bacteriome dysbiosis cannot predict *C. albicans* engraftment success

Altering the bacteriome with antibiotics did not consistently facilitate *C. albicans* colonization. We therefore decided to analyze the impact of the different antibiotics on (i) the total bacteria concentration throughout the experiment, (ii) the bacteriome composition at different timepoints during the distinct experimental periods (stabilization, antibiotic treatment, and/or recovery), and (iii) the bacterial metabolic activity (see Supplementary Information file for full time-course data on total bacterial concentrations, metabolite profiles, and raw metagenomics results).

First, the total bacteria concentration was constant throughout the baseline period for all bioreactors of both donors, and values remained largely unchanged in the control condition from both donors at ca. 9–10 log_10_(16S rRNA gene copy/mL) (Fig. S3). Interestingly, for donor A, clindamycin treatment had no impact on this level, whereas a strong reduction was observed upon ciprofloxacin and metronidazole treatments. By contrast, all antibiotics led to a strong reduction in bacterial concentrations in the microbiota derived from donor B. Those observations did not correlate with *C. albicans* engraftment success, suggesting that total bacterial load was not the main determinant of subduing *C. albicans*. We therefore more closely investigated shifts in bacteriome composition.

**Fig. 3:**
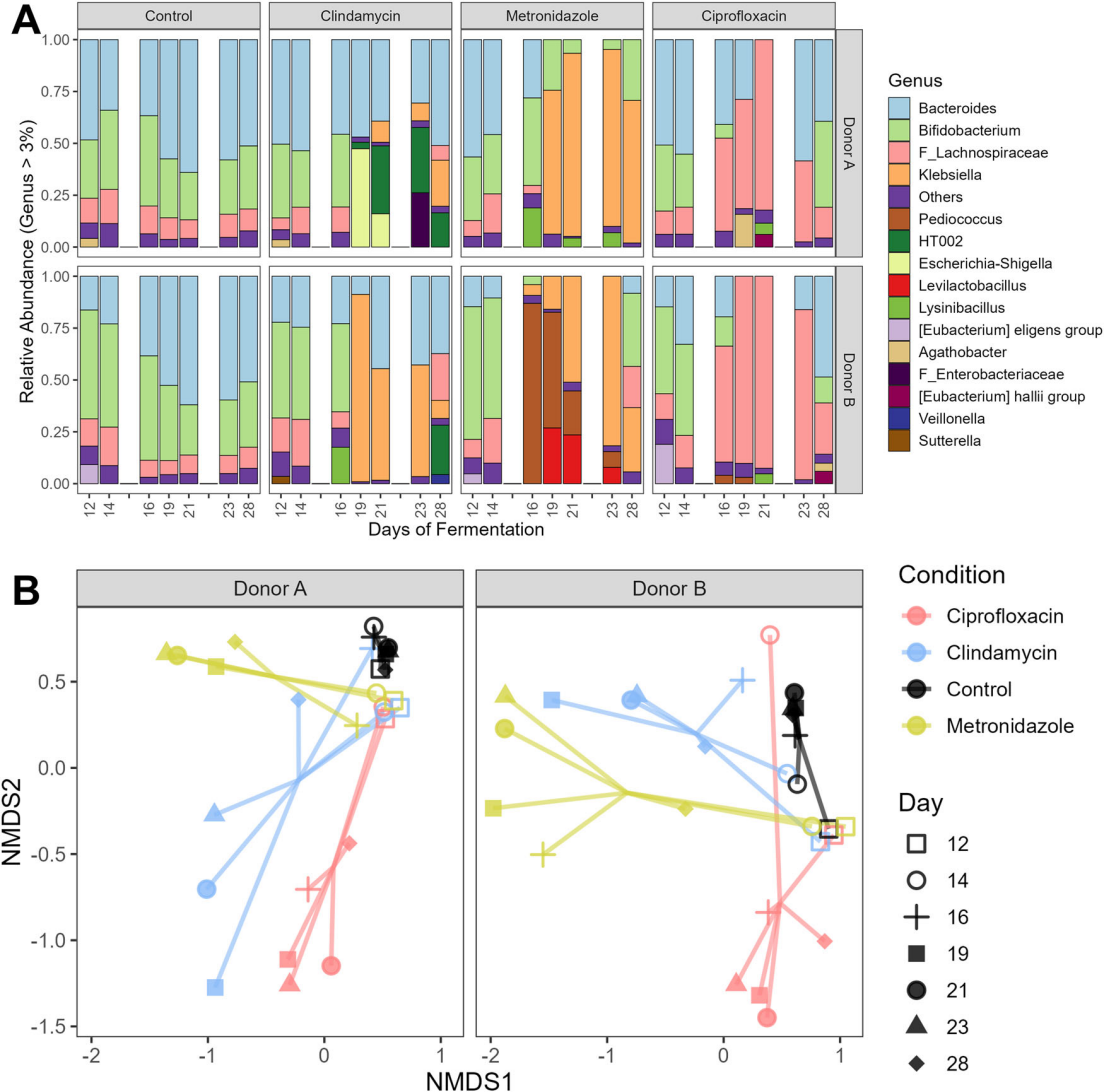
Microbiome permissiveness to *C. albicans* is not explained by overall taxonomic shifts, under simulated proximal colon physiological conditions. Bacteriome shift is antibiotic-dependent, as observed in the relative genus-level composition (**A**) and the resulting β-diversity (**B**). The impact of three antibiotics were tested in two donors: clindamycin, ciprofloxacin, and metronidazole, compared to a control. Despite similarities in community trajectories (e.g., metronidazole and clindamycin clustering for Donor B, or clindamycin and ciprofloxacin clustering for Donor A), only specific treatments led to microbiome permissiveness to *C. albicans*, as shown in Fig. 2. Samples were collected during the baseline period (days 12 and 14) during which *C. albicans* strain SC5314 was inoculated at days 0 and 2, during the antibiotic treatment (days 16, 19, and 21), which started with the re-inoculation of *C. albicans* on day 15, and during the recovery period (days 23 and 28). **B** Nonmetric multidimensional scaling (Bray-Curtis dissimilarity) of the genus-level relative bacteriome profiling. HT002 refers to “*Lactobacillus* sp. oral clone HT002”.

We found that the bacteriome composition was similar across all four bioreactors from a given donor during the baseline period (days 12 and 14) (Fig. 3A), and hence samples clustered together (Fig. 3B). Both donors were dominated, but with their own respective ratio, by *Bacteroides*, *Bifidobacterium*, and genera belonging to *Lachnospiraceae*. However, subsequent to the antibiotic treatment initiation, the bacteriome composition was strongly altered resulting in an increased abundance of distinct microbial taxa, including high levels of *Escherichia-Shigella*, *Klebsiella*, *Pediococcus*, *Lactobacillaceae* HT002, and *Levilactobacillus*. As a result of these changes, samples no longer clustered together, but per condition (Fig. 3B). In addition, the bacteriome α-diversity depicted microbial enrichment in both clindamycin- and ciprofloxacin-treated bioreactors, but not in metronidazole-treated ones from donor A (Fig. S4). Consequently, these observations suggest that the extent of bacteriome dysbiosis did not directly correlate with *C. albicans* colonization success, implying that specific bacterial taxa, rather than overall diversity or community disruption, may play a key role in mediating colonization resistance.

Given that overall microbial diversity did not account for differences in *C. albicans* persistence, we next examined microbial metabolic activity. Concentrations of short-chain fatty acids, lactate, and ethanol did not appear to correlate with the observed differences in engraftment success (Fig. S2). For instance, ethanol production was higher in most bioreactors upon antibiotic treatment (e.g., increased at day between 14% and 105% compared to the respective control), and this irrespective of *C. albicans* colonization. This observation suggests that the increase in ethanol was mainly associated with bacterial responses to antibiotic treatment, which is consistent with their higher overall cell counts compared to *C. albicans*. Furthermore, the clindamycin-impaired microbiomes from both donors exhibited high propionate levels and intermediate levels of acetate, butyrate, and lactate, which were comparable to those observed in microbiomes treated with other antibiotics where *C. albicans* failed to engraft. However, the limited number of donors may have hindered the identification of clear metagenomic and metabolic patterns. Therefore, follow-up experiments were conducted on additional donors, focusing exclusively on clindamycin.

### Clindamycin facilitates *C. albicans* engraftment across most tested donors

Clindamycin consistently facilitated *C. albicans* engraftment in the two tested donors A and B. To deepen our understanding of the bacterial shift that may underlie this mechanism, we subjected 12 additional human-derived microbiomes to clindamycin. Two *C. albicans* strains were used: SC5314 in experiment 3, similarly as in the previous experiments; and a GFP reporter strain of *C. albicans* SC5314 in the experiment 4. This reporter had no impact on the strain growth in the M-SHIME® in a microbiome-free environment (Figs. S2, S5), nor in mice (internal communication from Dr. Ilse Jacobsen, Hans Knöll Institute, Jena, Germany).

**Fig. 4:**
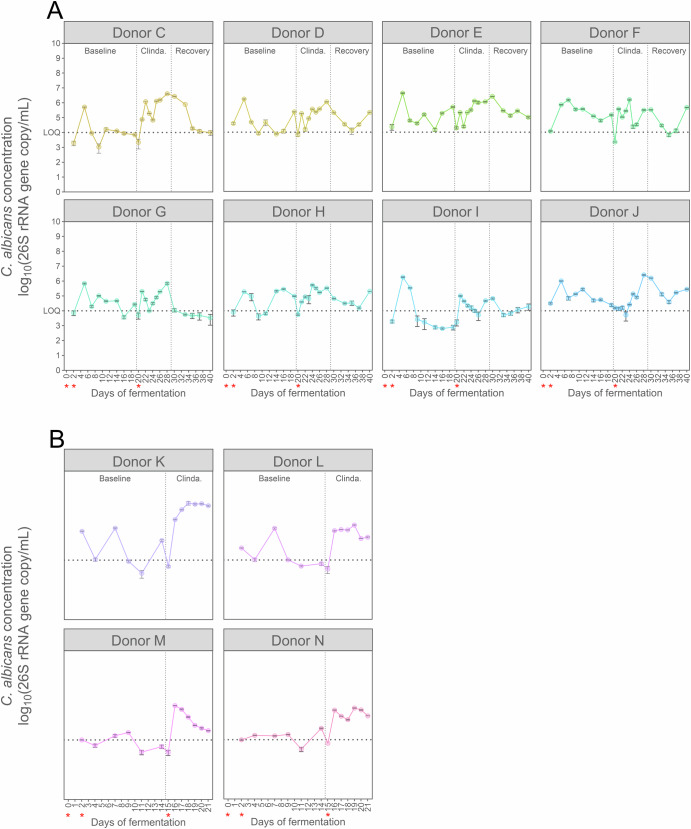
*Candida albicans* persistence and abundance is donor-dependent, and its concentration generally increases under clindamycin-treated condition. *C. albicans* concentration was measured by qPCR (*n* = 3) in presence of the faecal-derived microbiome from donor C to J (**A**) or K to N (**B**) and grown under simulated proximal colon physiological conditions. Data are represented as mean ± standard deviation. **A** Samples were collected in Experiment 3, specifically during the baseline period (days 0 to 19) during which *C. albicans* strain SC5314 was inoculated at days 0 and 2, throughout the clindamycin treatment (days 19 to 26; Clinda. = clindamycin), which started with the re-inoculation of *C. albicans* on day 20, and during the recovery period (days 26 to 40). **B** Samples were collected in Experiment 4, specifically during the baseline period (days 0 to 14) during which a GFP reporter strain of *C. albicans* SC5314 was inoculated at days 0 and 2, and throughout the clindamycin treatment (days 14 to 21), which started with the re-inoculation of *C. albicans* on day 15. *Candida albicans* inoculations are indicated by red asterisks. The limit of quantification ( = LOQ) is indicated by a dotted line. The antibiotic treatment period is represented by vertical dotted lines.

*Candida albicans* successfully persisted in the microbiome derived from 8 out of 12 donors during the baseline period, with values above the limit of quantification as assessed by qPCR (Fig. 4), and this was consistent with the calculated specific growth rates (Fig. S1). Nevertheless, concentrations remained generally low, below 5 log_10_(26S rRNA gene copy/mL) by the end of the baseline period. Supplementing clindamycin subsequently increased *C. albicans* concentrations in 10 out of 12 donors, while it remained unchanged in two donors (donors F and H) (Fig. 4). This demonstrated donor- and time-dependent dynamics, confirming the role of the microbiome in limiting *C. albicans* growth, and of clindamycin in suppressing it. Finally, stopping clindamycin treatment allowed the bacteriome to recover and this was concomitant with a substantial reduction in *C. albicans* concentration at most time points across all donors (Fig. 4).

### Clindamycin induces shifts in bacteriome composition without defining a *C. albicans*-permissive profile

During the baseline period, the total bacterial concentration ranged between 9 and 10.5 log_10_(16S rRNA gene copy/mL) for all donors (Fig. S3). While total bacteria concentration initially decreased in most donors upon initiation of clindamycin treatment, it returned to pre-treatment levels in subsequent days.

**Fig. 5:**
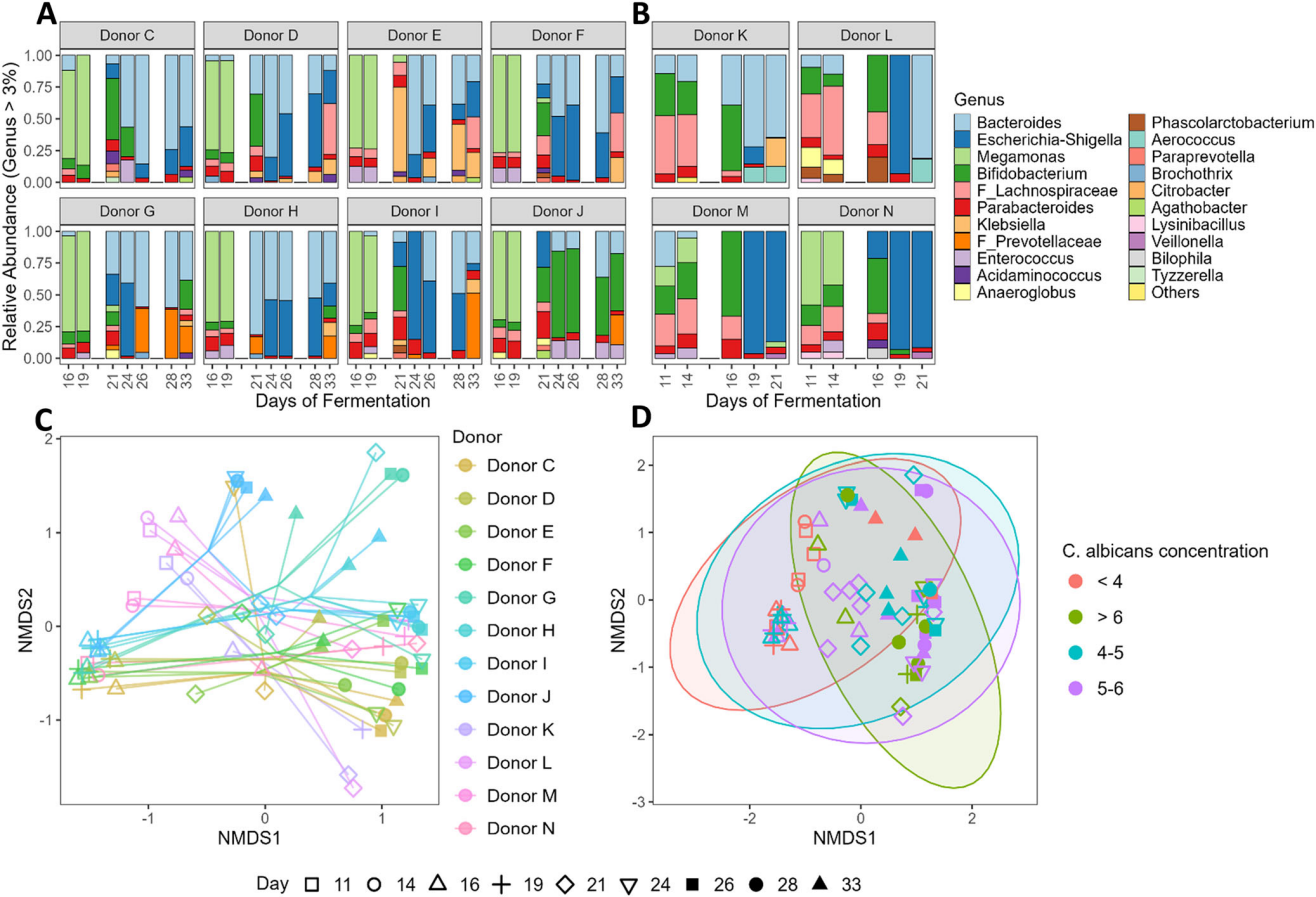
Clindamycin treatment alters the bacteriome composition across donors, but no specific profile predicts *Candida albicans* colonization success. Relative microbiome profiling from the simulated proximal colonic compartments inoculated with the faecal-derived microbiome from donors C to J (**A**), from donors K to N (**B**) and their resulting β-diversity (**C**, **D**), from samples collected during the baseline, antibiotic, and recovery periods. **A** Samples were collected in Experiment 3, specifically during the baseline period (days 16 and 19) during which *C. albicans* strain SC5314 was inoculated at days 0 and 2, during the clindamycin treatment (days 21, 24, and 26), which started with the re-inoculation of *C. albicans* on day 20, and during the recovery period (days 28 and 33). **B** Samples were collected in Experiment 4, specifically during the baseline period (days 12 and 14) during which a GFP reporter strain of *C. albicans* SC5314 was inoculated at days 0 and 2, and during the clindamycin treatment (days 16, 19, and 21), which started with the re-inoculation of *C. albicans* on day 15. **C**, **D** Nonmetric multidimensional scaling (Bray–Curtis dissimilarity) of the genus-level relative microbiome profiling, with a focus on either the donors (**C**), or the range of concentrations in *C. albicans*, expressed in log_10_(26S rRNA gene copy/mL) (**D**), with ellipses representing 95% confidence intervals.

Because total bacterial concentration did not explain differences observed between time points and donors, we analyzed the bacteriome genus level composition at various time points throughout the experiments (Fig. 5). Surprisingly, the bacteriome from donors C–J was dominated by *Megamonas* during the baseline period, a member of the family *Selenomonadaceae* which produces propionate (Fig. 5A). In contrast, the bacteriome from donors K–N displayed greater diversity (Fig. 5B). Regardless of the initial microbial diversity, the bacteriome composition shifted and α-diversity drastically decreased for all donors upon clindamycin treatment, and did not recover post antibiotic treatment (Fig. 5A, B, Fig. S4). Consequently, samples did not cluster per donor, nor per time point, suggesting various bacteriome profiles upon clindamycin treatment (Fig. 5C). Interestingly, samples did neither cluster in function of *C. albicans* concentration, suggesting that at a genus level and across donors, there was not one specific bacteriome profile that enabled *C. albicans* to grow or to be inhibited (Fig. 5D).

### Specific bacterial taxa, especially acetate and butyrate producers, negatively correlate with *C. albicans* concentration

**Fig. 6:**
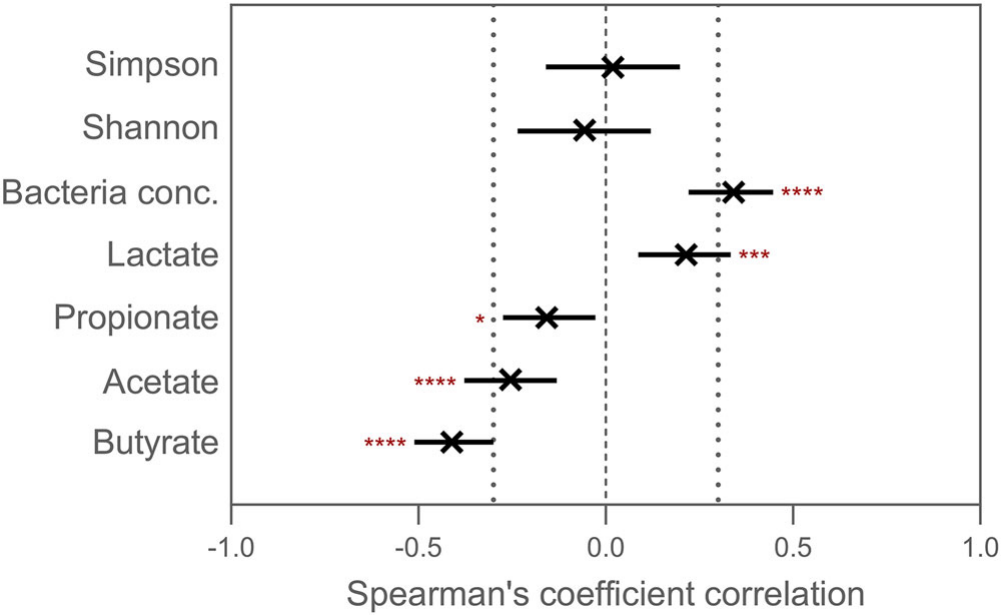
Butyrate and acetate are negatively correlated with *C. albicans* concentration. Data represent the Spearman’s ρ ± 95% confidence interval. Spearman’s correlation tests included samples taken between days: 5–12, and 19–30 (Donors A – B); 5–19, and 23–40 (Donors C – J); 7–14, and 18–21 (Donors K – N), for which the impact of *C. albicans* inoculation on its concentration in the bioreactors was expected minimal. The absolute value of the Spearman’s coefficient correlation was considered significant if higher than 0.3, and indicated on the figure with dotted lines. The *p*-values are indicated as follows: * < 0.1; *** < 0.001; **** < 0.0001.

To better understand the role of the healthy microbiome in preventing *C. albicans* engraftment, we first performed Spearman’s correlation analyses between *C. albicans* concentrations and bacteriome α-diversity metrics (Shannon and Simpson indices) and total bacterial concentrations (Fig. 6, Fig. S6). Data from the 14 clindamycin-treated bioreactors (donors A to N) were considered; however, only samples collected three days after *C. albicans* inoculation were included in the analysis. This filtering step was implemented to avoid potential bias from the inoculation event itself. Indeed, within the first two days following inoculation, *C. albicans* concentrations may reflect the residual inoculum rather than active growth. Interestingly, no significant associations were found between *C. albicans* colonization success and α-diversity. However, we observed a strong positive correlation with total bacterial load, suggesting that colonization resistance was not due to outnumbering *C. albicans*.

**Fig. 7:**
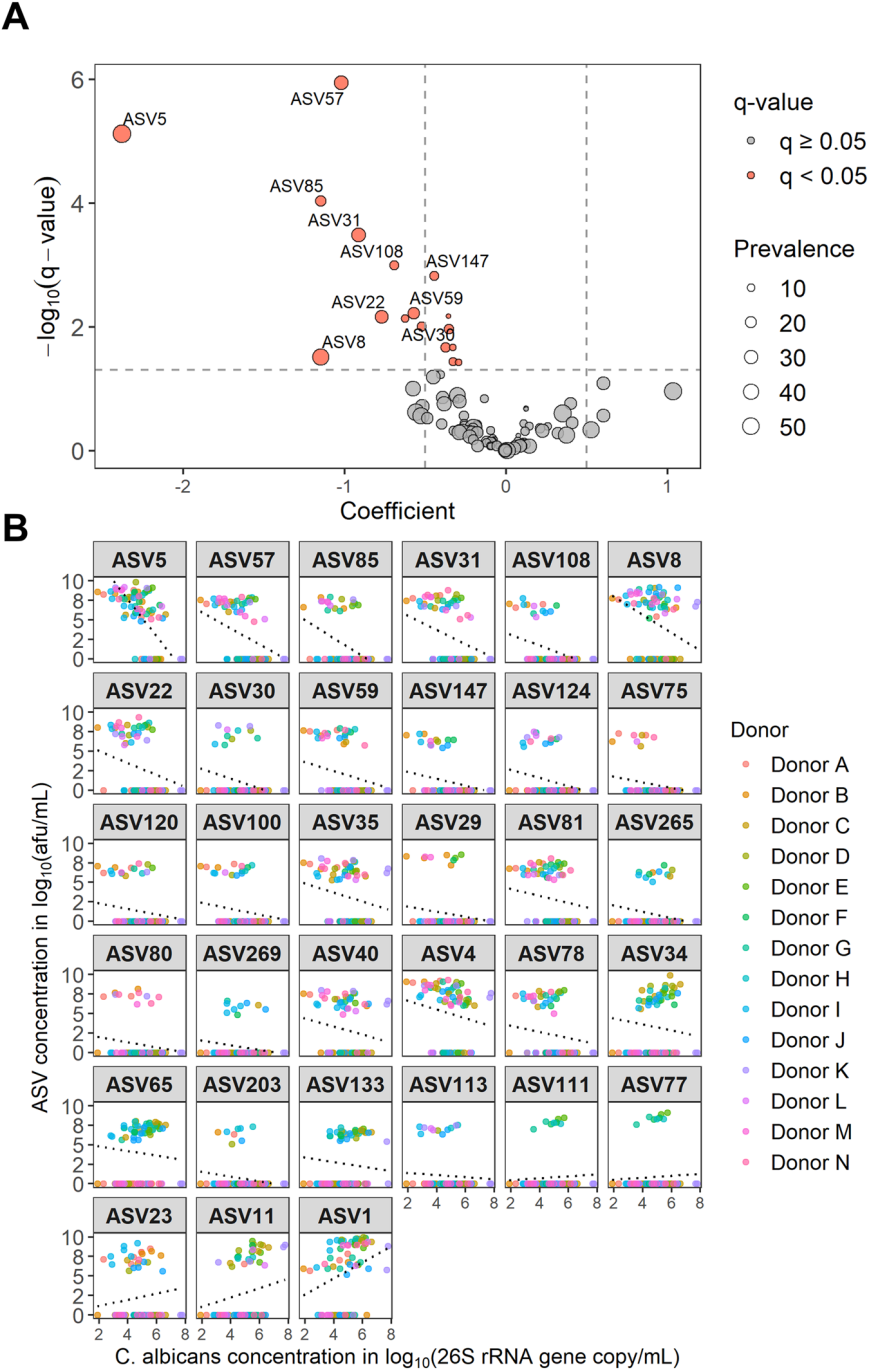
Association of ASV abundance with *C. albicans* concentration, assessed using MaAsLin2. The name of the ASVs can be found in Table 1. **A** Volcano plot showing the association between ASV abundance and *C. albicans* concentration. Point size reflects ASV prevalence across samples (*n* = 76), and color indicates statistical significance (*q*-value threshold of 0.05). The 10 ASVs with the largest effect size (calculated as –log_10_(*q*-value) × coefficient) are labeled. **B** Scatter plots showing *C. albicans* concentration (qPCR) in relation to corrected ASV abundance (relative abundance × total bacterial concentration by flow cytometry). The black dotted line shows the MaAsLin2-derived regression line (coefficient of association). Facets are ordered by effect size, from most negative to most positive (top left to bottom right) from the MaAsLin2 analysis.

Next, we investigated whether specific microbial taxa were associated with *C. albicans* levels using MaAsLin2, accounting for inter-individual donor variability and temporal dynamics (Table 1, Fig. 7, Fig. S7). This analysis revealed 28 bacterial taxa that were negatively associated with *C. albicans* concentration. The most strongly correlated ASVs belonged predominantly to the *Lachnospiraceae* family – known butyrate and acetate producers—including two unclassified genera (ASV5, present in 75% of samples; ASV22, 37%), *Agathobacter* (ASV31, 41%), *Blautia* (ASV57, 43%; ASV147, 17%), and *Fusicatenibacter* (ASV108, 17%). Additionally, negative associations were found with *Bifidobacterium* (ASV8, 62%; ASV59, 28%)—major acetate producers—and *Bacteroides* (ASV30, 13%)—acetate and propionate producers. In contrast, only four bacterial taxa showed positive correlations, and these were of relatively low effect size. Among them, *Escherichia/Shigella* (ASV1, 68%), a facultative anaerobe, showed the strongest association. Together, these results supported the hypothesis that *C. albicans* colonization was promoted more by the depletion of protective bacterial taxa than by the enrichment of permissive ones.

**Table 1:** Associations between microbial amplicon sequence variants (ASV) and *C. albicans* concentration

Phylum	Family	Genus	ASV	coef	stderr	n.not.0	n	*p*-value	*q*-value	Effect size
Actinobacteriota	*Bifidobacteriaceae*	*Bifidobacterium*	ASV008	−1.15	0.45	47	76	0.013	0.031	−**1.74**
			ASV059	−0.57	0.18	21	76	0.002	0.006	**−1.28**
			ASV035	−0.58	0.29	37	76	0.049	0.101	−0.58
			ASV040	−0.52	0.31	33	76	0.103	0.194	−0.37
			ASV004	−0.56	0.37	50	76	0.131	0.240	−0.35
	*Cellulomonadaceae*	*Oerskovia*	ASV133	−0.29	0.16	30	76	0.083	0.160	−0.23
	*Coriobacteriaceae*	*Collinsella*	ASV081	−0.45	0.20	33	76	0.030	0.064	−0.54
Bacteroidota	*Bacteroidaceae*	*Bacteroides*	ASV023	0.40	0.23	24	76	0.090	0.173	0.31
			ASV034	−0.38	0.22	34	76	0.091	0.174	−0.29
			ASV030	−0.63	0.20	10	76	0.002	0.007	**−1.33**
			ASV011	0.60	0.29	27	76	0.038	0.082	0.65
	*Prevotellaceae*	*F_Prevotellaceae*	ASV111	0.12	0.08	8	76	0.114	0.211	0.08
			ASV077	0.12	0.08	8	76	0.110	0.206	0.08
Firmicutes	*Aerococcaceae*	*Aerococcus*	ASV065	−0.30	0.16	43	76	0.063	0.128	−0.27
	*Butyricicoccaceae*	*Butyricicoccus*	ASV203	−0.31	0.17	8	76	0.076	0.149	−0.25
	*Lachnospiraceae*	*F_ Lachnospiraceae*	ASV005	−2.38	0.44	57	76	0.000	0.000	**−12.18**
		*Blautia*	ASV057	−1.02	0.17	33	76	0.000	0.000	**−6.08**
		*Agathobacter*	ASV031	−0.91	0.21	31	76	0.000	0.000	**−3.18**
		*Fusicatenibacter*	ASV108	−0.69	0.18	13	76	0.000	0.001	**−2.07**
		*F_Lachnospiraceae*	ASV022	−0.77	0.24	28	76	0.002	0.007	**−1.66**
		*Blautia*	ASV147	−0.44	0.12	13	76	0.000	0.002	**−1.25**
		*F_Lachnospiraceae*	ASV075	−0.36	0.11	8	76	0.002	0.007	−0.78
		*[Eubacterium] hallii group*	ASV120	−0.36	0.12	15	76	0.004	0.011	−0.70
		*[Eubacterium] hallii group*	ASV100	−0.38	0.14	15	76	0.009	0.021	−0.63
		*F_Lachnospiraceae*	ASV029	−0.33	0.12	9	76	0.009	0.021	−0.55
		*Roseburia*	ASV078	−0.39	0.21	25	76	0.069	0.138	−0.34
		*Tyzzerella*	ASV113	−0.13	0.07	11	76	0.073	0.145	−0.11
	*Veillonellaceae*	*Veillonella*	ASV080	−0.33	0.13	11	76	0.016	0.037	−0.47
Proteobacteria	*Enterobacteriaceae*	*F_Enterobacteriaceae*	ASV265	−0.41	0.18	11	76	0.027	0.060	−0.50
		*Citrobacter*	ASV269	−0.29	0.12	9	76	0.017	0.037	−0.42
		*Escherichia-Shigella*	ASV001	1.03	0.53	52	76	0.053	0.108	1.00
	*Sutterellaceae*	*Sutterella*	ASV124	−0.52	0.17	13	76	0.004	0.010	−1.05
		*Sutterella*	ASV085	−1.15	0.25	18	76	0.000	0.000	**−4.64**

The analysis included samples taken on days: 12,14, 19, 21, 23, 28 (Donors A – B); 16,19, 24, 26, 28, 33 (Donors C – J); 11, 14, 19, 21 (Donors K – N), for which the impact of C. albicans inoculation on its concentration in the bioreactors was expected minimal. Each ASV was selected at a prevalence of 10% across samples. Data was filtered at 0.01% abundance, normalized by applying total-sum scaling (TSS), and log-transformed. They were then regressed against C. albicans concentrations using Linear Models (LM). Taxa-C. albicans concentration associations with Benjamini & Hochberg false discovery rate (FDR) adjusted p-value (q-value) ≤ 0.3 are shown. The effect size was calculated as –log10(q-value) × coefficient. The n.not.0 number refers to the amount of samples containing detectable levels of the ASV across all samples (n=76). The top 10 negative effect sizes are indicated in bold.

To validate these findings at the functional level, we performed Spearman’s correlation analyses between *C. albicans* concentrations and metabolite concentrations (acetate, propionate, butyrate, and lactate) (Fig. 6, Fig. S6). While lactate (positive) and propionate (negative) exhibited only modest associations, both acetate and, in particular, butyrate displayed strong negative correlations with *C. albicans* levels. These metabolic correlations perfectly aligned with the taxonomic associations observed previously, i.e., the depletion in *Lachnospiraceae* and *Bifidobacterium* concomitant with increased *C. albicans* concentrations.

## Discussion

Treating candidiasis may involve restoring colonization resistance by reestablishing the initial microbiome composition and/or functionality^25^. To effectively restore colonization resistance, it is essential to identify the specific microbes that contribute to keeping *C. albicans* at bay. This can be done using the M-SHIME® model: being devoid of host-microbiome interactions, it provides insights into *C. albicans*-microbiome dynamics from an ecological standpoint.

Our study confirmed that the physiological conditions of the colon support the growth of *C. albicans*. Indeed, *C. albicans* thrived in a microbiome-free environment, devoid of colonization resistance, reaching concentrations exceeding 8 log_10_(26S rRNA gene copy/mL), similar to those observed in germ-free or penicillin-treated mice^22^. When gut microbes were present, *C. albicans* enrichment primarily occurred in microbiomes dominated by a limited number of taxa (*Megamonas*, *Escherichia*/*Shigella*, or *Klebsiella*) or compromised by certain antibiotics. More specifically, while clindamycin consistently facilitated *C. albicans* colonization across different microbiome backgrounds (12/14 donors), metronidazole (1/2) and ciprofloxacin (0/2) did not. Our findings align with human and mouse studies showing the limited impact of ciprofloxacin on *C. albicans*^26–29^, and the stronger impact of clindamycin compared to metronidazole in mice^22^. Despite inducing long-lasting changes in microbial diversity^30,31^, ciprofloxacin is associated with a lower rate of common gastrointestinal side effects as compared to other antibiotics^32^, which could be attributed to sustained colonization resistance. Moreover, to our knowledge, reports suggesting candidiasis as a side effect of ciprofloxacin are scarce^33–35^, confirming a lower risk of *C. albicans* opportunistic blooms associated with ciprofloxacin use. However, as only two donors were included in this comparison, further experiments are needed to confirm the differential capacity of each antibiotic to impair colonization resistance against *C. albicans*.

Because the three antibiotics led to distinct *C. albicans* colonization outcomes, we first assessed whether colonization success correlated with total bacterial concentration. Interestingly, total bacterial concentration was positively associated with *C. albicans* levels, suggesting that colonization resistance is not due to outnumbering *C. albicans*. We therefore investigated whether specific bacterial taxa were responsible for colonization resistance, considering two factors: (i) different antibiotics have distinct antibacterial properties targeting specific bacterial taxa, and (ii) different donors respond differently to antibiotic treatment. For instance, faecal *C. albicans* concentrations upon β-lactam antibiotic treatment were previously reported to be quite variable between individuals. Lower activity of endogenous faecal β-lactamase, which inhibits the antibiotic’s efficacy, correlated with more changes in microbial composition and consequently higher *C. albicans* levels^36^.

Using 16S rRNA gene-targeted Illumina sequencing on samples from donors A and B bioreactors, we found that clindamycin and metronidazole induced an increase in *Enterobacteriaceae* (*Escherichia*/*Shigella*, *Klebsiella*) and a reduction in *Lachnospiraceae* and *Bifidobacteriaceae* (*Bifidobacterium*), consistent with previous in vivo research^37^. In contrast, ciprofloxacin-induced dysbiosis strongly increased *Lachnospiraceae*. Interestingly, *Lachnospiraceae* decreased during the washout of ciprofloxacin, which was concomitant to increased *C. albicans* levels. Taken together, these results suggest that *Bifidobacteriaceae* and *Lachnospiraceae* contribute to the colonization resistance against *C. albicans*. To validate these findings, we extended the analysis to include 12 additional donors, focusing specifically on clindamycin treatment. Using MaAsLin2^38^, we confirmed significant negative associations between *C. albicans* concentrations and 28 bacterial taxa, with the most pronounced effects observed for members of the *Lachnospiraceae* family, *Bifidobacterium*, and *Bacteroides*.

These taxonomic associations were further supported by metabolite analysis. Specifically, *C. albicans* engraftment was negatively correlated with butyrate and acetate concentrations, as revealed by Spearman’s correlation tests. These findings are consistent with the depletion of *Lachnospiraceae* and *Bifidobacterium*, which are primary producers of butyrate^39^, and acetate^40^ in the human gut, respectively. In fact, the inhibitory role of *Bifidobacterium* species against *C. albicans* is supported by several studies across in vitro^41^, in silico^42^, in mice^43^, and human supplementation studies^44^. Similarly, previous reports correlated *C. albicans* colonization with the loss of specific *Lachnospiraceae* species^45^. Notably, a recent study demonstrated that antibiotic-mediated depletion of Clostridia, which includes *Lachnospiraceae*, reduces butyrate production leading to epithelial oxygenation, thereby disrupting the mucosal hypoxic environment, and promoting *C. albicans* growth^46^. In our study, butyrate could not induce hypoxia via β-oxidation by colonocytes due to the absence of a host component in the M-SHIME®. Yet, its reduction, along with acetate reduction, indicates diminished anaerobic metabolism. This shift increases the redox potential of the simulated intestinal suspension, making oxygen more available for facultative anaerobes, including *C. albicans* and *Escherichia/Shigella*. Indeed, one ASV related to *Escherichia/Shigella* (ASV1) was positively correlated with *C. albicans* levels, likely reflecting a shared ecological advantage under dysbiotic, oxygen-enriched conditions. Although speculative, this may promote *C. albicans* growth, however, further research with monitoring of redox potential is required to confirm this hypothesis.

Besides being markers of increased redox potential, microbiota-derived SCFAs have direct impacts in suppressing *C. albicans* colonization (reviewed recently by McCrory et al.^47^). In fact, Guinan et al. showed that broad-spectrum antibiotic treatment in mice led to reduced levels of cecal acetate, propionate, and butyrate, which coincided with increased *C. albicans* burden^48^. Furthermore, they showed that physiological concentrations of these SCFAs inhibited fungal growth in RPMI 1640 medium. Building on this work, our study investigated these interactions under defined, reproducible, and host-independent conditions, using microbiota derived from 14 human donors. Our approach offers several advantages. First, by including a diverse panel of donors, we captured inter-individual variability in colonization resistance, identifying distinct susceptibilities to *C. albicans* overgrowth and associated shifts in SCFA profiles. Second, by applying different antibiotics to the same donor-derived microbiota, we directly compared how each compound altered specific SCFA-producing taxa and facilitated *C. albicans* expansion. Third, the time-resolved sampling strategy enabled us to temporally align microbiome disruption, SCFA depletion, and *C. albicans* colonization dynamics.

An alternative putative role of *Lachnospiraceae* in inhibiting *C. albicans* colonization may involve converting primary bile acids to secondary bile acids through dehydroxylation, as observed with *L. scindens* (formerly *Clostridium scindens*)^49^. Indeed, primary bile acids were shown to enhance *C. albicans* virulence and growth^50–52^, whereas secondary bile acids to inhibit these processes both in mice and in vitro^51,53^. However, the resolution at genus level of our sequencing approach could not confirm the presence of secondary bile acid converters. To address this, we performed a pathway prediction analysis using Tax4Fun2^54^, which revealed no significant correlations between the primary and secondary bile acid biosynthesis pathways and *C. albicans* outgrowth (*p* = 0.38 and 0.15, respectively, Fig. S8). Although previous studies have reported opposing effects of primary and secondary bile acids on *C. albicans*, our findings suggest that other factors, such as butyrate and acetate reduction, played a more dominant role in shaping a niche favorable for *C. albicans* growth.

In contrast to the negative correlation with acetate and butyrate, lactate positively correlated with *C. albicans* levels, as observed in Spearman’s correlation tests. While lactate inhibits *C. albicans* virulence and growth in the vaginal milieu^55,56^ and is beneficial for vaginal health, its accumulation in the human colon indicates bacterial dysbiosis, often observed with inflammatory bowel diseases such as ulcerative colitis or Crohn’s disease^57^. In the colon, lactate accumulation is the symptom of an incomplete bacterial cross-feeding network, in which lactate is an important precursor to producing e.g., propionate^39^. Furthermore, the pKa of lactate (3.86), lower than the one of propionate (4.75), acetate (4.76), and butyrate (4.91)^58,59^, results in a lower antimicrobial effect at the prevailing pH of the proximal colon (*in casu* controlled in the M-SHIME® between 5.75 and 5.95).

Only five of the 33 bacterial taxa identified by MaAsLin2 showed positive correlations with *C. albicans* concentrations. In our view, this further supports the hypothesis that *C. albicans* colonization is facilitated more by the depletion of protective bacterial taxa than by enrichment of permissive ones. Among these positively correlated taxa, two ASVs belonged to *Bacteroides* (ASV23, ASV11), whereas, as mentioned earlier, other *Bacteroides* ASVs were negatively correlated. *Bacteroides* are known for their fundamental role in breaking down complex carbohydrates, including mannan^60^. Notably, mannan is abundant on *C. albicans* cell surface^61^. Using co-culture, it was hypothesized that *C. albicans* mannan acts as a substrate for *Bacteroides* to grow^62^. Altogether, this could explain the strong negative correlations observed in our study. However, by breaking down mucin into smaller carbohydrates, *Bacteroides* supplementation in mucin-containing medium was also associated with higher *C. albicans* growth^63^, suggesting a positive symbiosis as also observed in our analysis.

Overall, our study indicates that certain ASVs producing acetate and butyrate, belonging to *Bifidobacterium*, *Bacteroides*, and *Lachnospiraceae* species, present in most, if not all, donors who participated, emerge as potential modulators of *C. albicans* growth, either acting individually or collectively. Yet, further investigations are required considering the limitations of this study. Despite being supported by other reports both in vivo and in vitro, our experiments involved a limited number of experimental repetitions. By definition, our in silico analysis can only give partial insights; supportive intervention studies are therefore required. In addition, the host component, absent in this ecological study, may have a strong contribution in the microbial interplays and in selecting the microbes that can engraft the microbiome as well as in controlling their level. In particular, the system lacks host components such as intestinal epithelium, mucosal immune responses, and neuroendocrine signaling. Consequently, it does not capture the influence of host-mediated antimicrobial defenses, inflammation, or epithelial oxygenation, which can shape *C. albicans* colonization and pathogenicity in vivo. Additionally, antibiotics in this model act solely on the microbial community and do not reflect systemic effects or host responses that might indirectly affect fungal colonization. Therefore, while the model is well suited for studying ecological mechanisms of colonization resistance, its findings should be interpreted as complementary to in vivo models where host–fungus interactions play a central role.

## Methods

### Chemical products

All chemicals were obtained from Merck (Darmstadt, Germany) unless otherwise stated.

### In vitro M-SHIME® technology

The M-SHIME® technology (ProDigest and Ghent University, Ghent, Belgium), described by Van den Abbeele and colleagues^64^, was adapted to only simulate the combined stomach-small intestine and the proximal colon. The proximal colon compartments (250 mL) were continuously homogenized through stirring, and pH was controlled between the range of 5.75 to 5.95. Every reactor was maintained at 37 °C and kept anaerobic by flushing the headspace with nitrogen (N_2_), once per day. Three times per day, the combined stomach-small intestine compartment received 70 mL fresh nutritional medium and 30 mL pancreatic juice per connected proximal colon compartment. After 1.5 h of incubation, the mixture was transferred into the proximal colon compartments. The M-SHIME® feed mimicked the colonic nutrient composition of adults, and contained (in g/L) arabinogalactan (1.2), pectin (2.0), xylan (0.5), starch (4.0), glucose (0.4), yeast extract (3.0), peptone (1.0), mucin (2.0) and cystein (0.5). The pancreatic juice contained (in g/L) NaHCO_3_ (12.5), bile salts (6.0) (Difco, Bierbeek, Belgium) and pancreatin (0.9). To simulate the mucosal component of the human gastrointestinal tract, mucin-covered microcosms (length = 7 mm, diameter = 9 mm, total surface area per m^3^ = 800 m^2^, AnoxKaldnes K1 carrier, AnoxKaldnes AB, Lund, Sweden) were introduced in the proximal colon compartments. Their preparation and replacement was performed as previously described^64^. Finally, the proximal colon compartments were inoculated (5% V/V) at day 0 with the faecal sample of a healthy donor.

### *Candida albicans* growth in a microbiome-free environment under physiological conditions simulating the human proximal colon (Experiment 1)

**Fig. 8:**
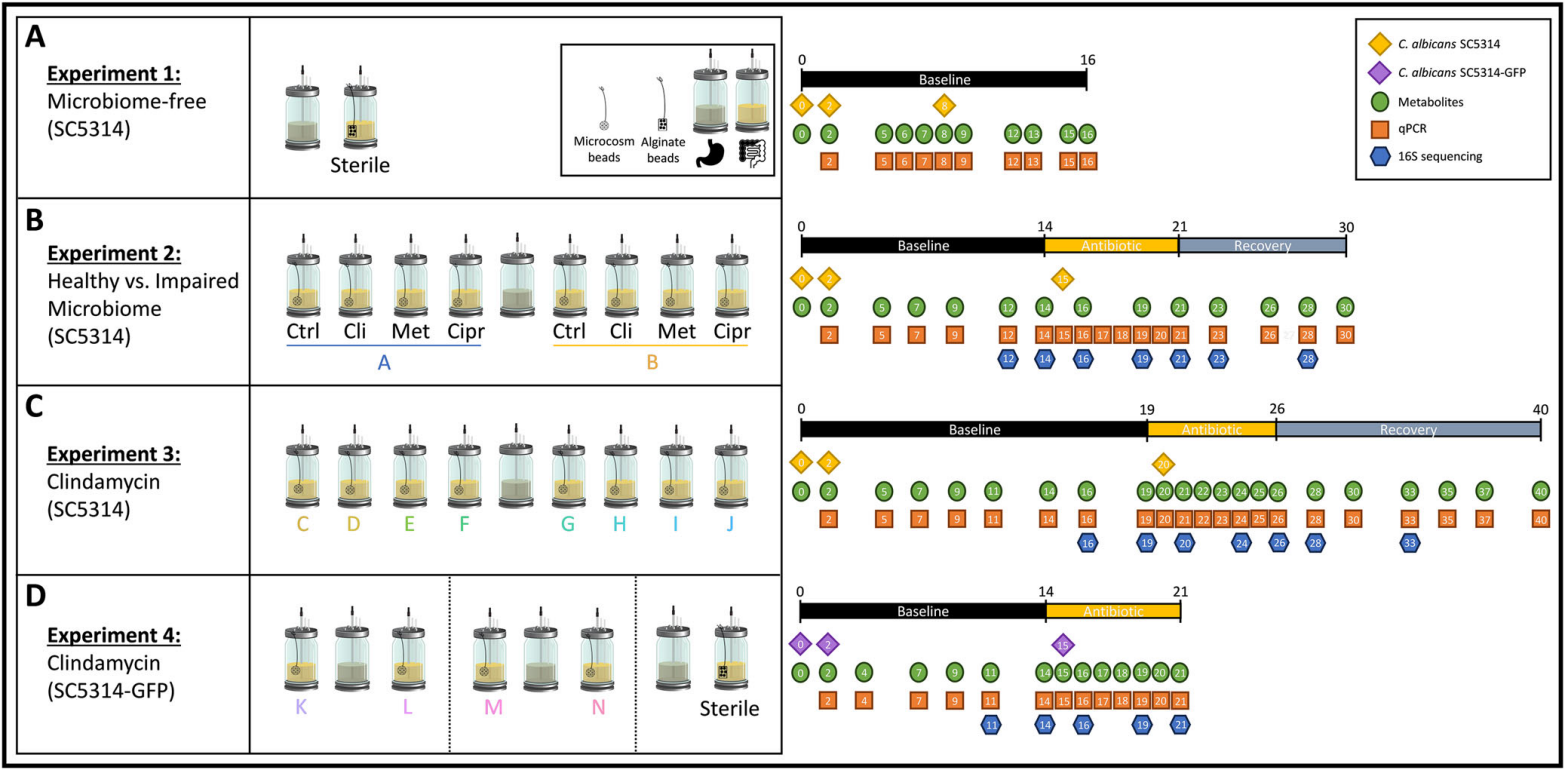
Experimental design of the long-term M-SHIME®. **A** *Candida albicans* strain SC5314 was inoculated in a microbiome-free bioreactor to assess its capacity to grow under simulated proximal colon physiological conditions. **B** *Candida albicans* strain SC5314 was inoculated in presence of the faecal-derived microbiome from donors A or B. Following a baseline period, bioreactors were dosed with either clindamycin, metronidazole, or ciprofloxacin, and their impact compared to a control bioreactor. *Candida albicans* strain SC5314 was reinoculated on day 15 to ensure its presence in the bioreactors regardless of its initial capacity to engraft during the baseline period. The competitive ability of *C. albicans* against a recovering microbiome was assessed following antibiotic cessation. Repetition of the antibiotic experiment was performed using the faecal-derived microbiome from donors C to J (**C**), or donors K to N (**D**), focusing on clindamycin only. While *C. albicans* strain SC5314 was used in (**C**), a GFP reporter strain of *C. albicans* SC5314 was used in (**D**). The absence of effect of the added reporter was pre-emptively confirmed in a microbiome-free environment. Ctrl control, Cli clindamycin, Met metronidazole, Cipr ciprofloxacin.

In a first experiment, the capacity of *C. albicans* to grow under simulated proximal colon physiological conditions in the absence of a microbiome background was evaluated (Fig. 8A). For this purpose, 1 mL of *C. albicans* strain SC5314 (see preparation details below) was inoculated on day 0 into an autoclaved (121 °C, 20 min) SHIME® reactor. The inoculation was repeated after sampling the SHIME® at days 2 and 8. Instead of mucin-covered microcosms as described above, which cannot be sampled in a sterile manner, the SHIME® reactor included mucin-alginate beads to simulate the mucosal component of the human gastrointestinal tract, as previously described^65^. In short, these mucin-alginate beads were prepared by dripping a mucin-alginate solution into a cross-linking solution containing CaCl_2_^65^. The SHIME® feed contained gentamicin (25 mg/L), penicillin G (21.6 mg/L) and streptomycin (100 mg/L) to ensure the sterility of the bioreactor throughout the 16-day experiment. Concentrations of *C. albicans*, short-chain fatty acids (SCFA), Branched Short-Chain Fatty Acid (BSCFA), lactate, and ethanol were quantified at various time points during the experiment (Fig. 8A). Samples were collected before the initiation of a new feeding cycle, thus during the stationary phase of *C. albicans* growth.

### *Candida albicans* growth in healthy and impaired faecal-derived microbiome environments (Experiment 2)

In a second experiment, the capacity of *C. albicans* to grow in simulated proximal colon conditions inoculated with faecal-derived microbiota was assessed (Fig. 8B). Briefly, faecal samples from two healthy donors A and B were used to inoculate four distinct proximal colon compartments per donor on day 0, along with 1 mL of *C. albicans* strain SC5314 (see preparation details below). *Candida albicans* was reinoculated after sampling the SHIME® at day 2 to account for potential inhibitory effects (i.e., the high microbial metabolite production and cell concentration characterizing the first hours following faecal inoculation). The experimental timeline comprised three distinct periods. First, the baseline period (days 0 to 14), during which the microbial community composition remained unaltered, provided insight into *C. albicans* growth capacity under conditions of eubiosis. During the second period (days 14 to 21), three proximal colon compartments per donor received three daily doses of antibiotics for seven days (see preparation details below), with either clindamycin (final concentration in the reactor 85 mg/L), metronidazole (85 mg/L) or ciprofloxacin (49 mg/L), while no antibiotic was administered in the fourth reactor ( = control). *Candida albicans* was reinoculated after sampling the SHIME® at day 15 to ensure its presence in the reactor, irrespective of its capacity to grow during the baseline period. Hence, the antibiotic period provided insights into *C. albicans’* growth capacity in differentially impaired faecal-derived microbiome backgrounds. Following the antibiotic cessation, the recovery of the microbial ecosystem was observed (days 21 to 30). This period aimed to assess the competitive ability of *C. albicans* against a recovering colonic microbiome. Concentrations of *C. albicans*, SCFA, BSCFA, lactate, and ethanol were quantified, along with the bacteriome community composition at various time points throughout the experiment (Fig. 8B). Samples were collected before the initiation of a new feeding cycle, thus during the stationary phase of microbial growth.

### *Candida albicans* growth in multiple healthy and clindamycin-impaired faecal-derived microbiome environments (Experiment 3 and 4)

In a third and a fourth experiment, observations from the clindamycin-impaired microbiome experiment were validated by repeating the conditions with faecal-derived microbiome backgrounds from additional donors (Fig. 8C, D). Indeed, clindamycin, but not the other antibiotics, consistently facilitated *C. albicans* engraftment in the two tested donors A and B. These findings prompted us to deepen our understanding of the bacterial shift that may underline this mechanism.

During the third experiment, faecal samples from donors C-J were used to inoculate distinct SHIME® reactors simulating the proximal colon. In addition, *C. albicans* strain SC5314 was inoculated on day 0, after sampling the SHIME® at day 2 during the baseline period, and after sampling the SHIME® at day 20 during the antibiotic treatment (see preparation details below). The experiment followed a similar approach to experiment 2, with, however, extended baseline (days 0 to 19) and recovery (days 26 to 40) periods. The microbiome was subjected to clindamycin (85 mg/L, final concentration) during the antibiotic period (days 19 and 26).

The timeline for the fourth experiment was similar to the third experiment, with clindamycin (85 mg/L, final concentration) administered for seven days (days 14 to 21) following a baseline period (days 0 to 14) to the simulated proximal colon compartments which were inoculated on day 0 with faecal-derived microbes from donors K-N. However, no recovery period was studied during this experiment. Furthermore, a GFP reporter strain of *C. albicans* SC5314 was used instead of the parental SC5314 strain, and 1 mL inoculated on day 0, after sampling the SHIME® at day 2 during the baseline period, and at day 15 during the antibiotic treatment (see preparation details below). This GFP reporter strain was used in view of a parallel research project to facilitate visualization. However, the reporter had no impact on viability in mice (internal communication from Dr. Ilse Jacobsen, Hans Knöll Institute, Jena, Germany). Strain viability was also assessed in the present study using a sterile SHIME® reactor whereby physiological conditions simulating the proximal colon were applied, as executed during the first experiment.

Concentrations of *C. albicans*, SCFA, BSCFA, lactate, and ethanol were quantified, along with the bacteriome community composition at various time points throughout both experiments (Fig. 8C, D). Samples were collected before the initiation of a new feeding cycle, thus during the stationary phase of microbial growth.

### *Candida albicans* inoculum preparation and inoculation

*Candida albicans* strain SC5314 (isolated from a patient with disseminated candidiasis)^66^ was provided by the research group of Dr. Salomé LeibundGut-Landmann (University of Zürich, Zürich, Switzerland). The *C. albicans* SC5314-GFP reporter strain was provided by the research group of Dr. Bernhard Hube (Leibniz-HKI, Jena, Germany). It had been constructed as previously described in Westman et al.^67^.

*Candida albicans* strain SC5314 was initially cultured on Sabouraud dextrose agar (Avantor™) containing gentamicin (50 µg/mL), and incubated for 48 h under aerobic conditions, at 37 °C. Subsequently, a single colony was selected to inoculate 50 mL of simulated batch proximal colon medium in a sterile Erlenmeyer. The Erlenmeyer was closed with a cotton plug, and placed in a UV-decontaminated 2.5 L anaerobic jar (Merck), and incubated at 37 °C, shaken at 70 rpm, under microaerophilic conditions using a CampyGen^TM^ (ThermoFischer Scientific, Waltham, MA, USA) bag, for 24 h. Subsequently, 1 mL of the culture was used to inoculate each simulated proximal colon compartments, at days as indicated in Fig. 8A–C. The simulated proximal colon contained (in g/L) arabinogalactan (0.96), pectin (1.64), xylan (0.4), starch (3.28), glucose (0.32), yeast extract (2.44), peptone (0.8), mucin (1.64) and was buffered with KH_2_PO_4_ (0.1 mM) and K_2_HPO_4_ (0.1 mM) at pH 5.9.

The GFP reporter strain of *C. albicans* SC5314 was initially grown on Yeast Peptone Dextrose (YPD, Carl Roth, Mannheim, Germany) agar containing 80 µg/mL chloramphenicol (Carl Roth) and 100 µg/mL ampicillin (Carl Roth), incubated at 30 °C, aerobically, for 48 h. Subsequently, a single colony was selected to inoculate 50 mL of YPD broth, and incubated at 30 °C, shaken at 120 rpm, aerobically, for 24 h. Finally, 1 mL of the culture was used to inoculate each simulated proximal colon compartments, at days as indicated in Fig. 8D.

The concentration of each inocula is detailed in Fig. S9. In all cases, the headspace of the proximal colon compartments was flushed with N_2_ immediately after inoculation.

### Antibiotic preparation

Clindamycin hydrochloride (3.15 g/L) and metronidazole (3.15 g/L) stock solutions were prepared in demineralized water and ciprofloxacin (1.81 g/L) stock solution in 0.1 M HCl, twice per week. The solutions were pumped automatically into the proximal colon compartments every 8 h, 30 min after the feed entered, at a flow rate of 1 mL/min for 2.7 min. The antibiotics reached their maximum concentration in the proximal colons by day four of administration, at 85 mg/L for clindamycin and metronidazole and 49 mg/L for ciprofloxacin.

### Faecal sample collection and donor description

The 14 healthy adults that donated a faecal sample had no antibiotic nor pre- or probiotic dietary supplements intake in the 3 months preceding the collection, did not follow special diets, had no disorders, and had no constipation. All faecal samples were immediately transferred to a recipient containing an AnaeroGen™ (ThermoFischer Scientific) bag to limit the samples’ exposure to oxygen, and kept in a fridge. The proximal colon compartments were inoculated within maximum 16 h post collection of the faecal samples by the donors. None of the faecal samples contained quantifiable levels of *Candida albicans* by qPCR, although this was not a selection criterion for inclusion in the study (Supplementary Information file).

The study was conducted in accordance with the Declaration of Helsinki and approved by the Ethics Committee of the University Hospital Ghent (reference number B670201836585). Informed consent of donors was obtained after providing them with detailed information about the project and the use of the samples.

### Metabolic analysis

To assess the impact of the gut microbiome metabolic activity on *C. albicans* colonization, lactate, ethanol, SCFA (acetate, propionate, and butyrate), and BSCFA (isobutyrate, isovalerate, and isocaproate) were quantified at the time points described in Fig. 8. Their quantification was performed as previously described^21^. All analyses were performed in simplicate.

### Microbial community analysis by Amplicon sequencing

The 16S rRNA gene-targeted Illumina sequencing data were processed using the DADA2 R package according to the pipeline instructions^68^. In a first quality control step, the primer sequences were removed, and reads were truncated at a quality score cut-off (truncQ = 2). Besides trimming, additional filtering was performed to eliminate reads containing any ambiguous base calls or reads with high expected errors (maxEE = 2.2). After dereplication, unique reads were further denoised using the Divisive Amplicon Denoising Algorithm (DADA) error estimation algorithm and the selfConsist sample inference algorithm (with the option pooling=TRUE). The obtained error rates were inspected and, after approval, the denoised reads were merged. Finally, the Amplicon Sequence Variant (ASV) table obtained after chimera removal was used for taxonomy assignment using the Naive Bayesian Classifier and the DADA2 formatted Silva v138^69^. Only sequences having at least 5 counts in one sample and assigned to the bacteria kingdom were retained. A total of 4524666 withheld sequences were binned into ASV.

### Microbial community analysis by qPCR

Total bacterial and *Candida albicans*-specific concentrations were determined by qPCR at the time points described in Fig. 8, and on the different *C. albicans* inocula. Briefly, DNA was isolated as previously described^70^ with minor modifications^71^ from 1 mL luminal samples or inocula. Subsequently, qPCR was performed using a QuantStudio 5 Real-Time PCR system (Applied Biosystems, Foster City, CA, USA). Each sample was run in technical triplicate.

Total bacterial concentration determination was performed as previously described^20^ with the primers UNI-F (5’-GTGSTGCAYGGYYGTCGTCA-3’) and UNI-R (5’-ACGTCRTCCMCNCCTTCCTC-3’)^72^, which target the 16S rRNA gene. Results are reported as log_10_(16S rRNA gene copies/mL).

*Candida albicans* quantification was performed as previously described^21^ using the primers Calb-F (5’-GGGTTTGCTTGAAAGACGGTA-3’) and Calb-R (5’-TTGAAGATATACGTGGTGGACGTTA-3’), and the probe Calb-P (5’-FAM-ACCTAAGCCATTGTCAAAGCGATCCCG-3’)^73^, which target the 26S rRNA gene. Results are reported as log_10_(26S rRNA gene copies/mL).

The standards used were synthesized gBlock Gene Fragments (Integrated DNA Technologies, Inc., Coralville, IA, USA) of 1000 bp designed by aligning the primer pairs on the reference genome of the *C. albicans*, which was retrieved from NCBI (Bethesda, MD, USA). For the total bacteria standard, *Escherichia coli* strain 97–3250 was used. The complete sequence of each standard can be found in Marsaux et al.^21^.

### Data analysis

In the case of total bacterial and *C. albicans* concentrations, data were log-transformed because of their log-normal distribution.

The specific growth rate of *C. albicans* was estimated to get insight into its capacity to grow under the applied physiological conditions and environmental stress. It was determined using the volume of fresh medium entering the proximal colon compartment, as previously described^21^. Briefly, the number of cells produced during each feeding cycle was first estimated (1).

1 $$P=\frac{\left[Cf\right]-[Ci]}{dt}$$

Where:

P = number of cells per mL produced during one feeding cycle between time f and time i

dt = considered time frame of cell production (i.e., 1 cycle = 8 h)

[Cf] = concentration of *C. albicans* at the end of the cycle (afu/mL)

[Ci] = concentration of *C. albicans* at the beginning of the cycle (afu/mL)

However, *C. albicans* concentration was only measured at specific days, with each day containing three feeding cycles. Thus, its concentration at the end of each feeding cycle between two time points of sampling was unknown. These unknown values were estimated by linearly extrapolating the measured values (2).

2 $$\left[Cu\right]=\left[Ct\right]+\frac{u}{n}\left(\left[CT\right]-[Ct]\right)$$

Where:

[Cu] = unknown concentration *C. albicans* at the end of the feeding cycle u between time points t and T (afu/mL)

[Ct] = measured concentration of *C. albicans* at the end of the feeding cycle t (afu/mL)

[CT] = measured concentration of *C. albicans* at the end of the feeding cycle T (afu/mL)

u = number of the feeding cycle

n = total number of feeding cycles between time points t and T

Having *C. albicans* concentrations at the end of each cycle throughout the entirety of the experiment, it is then possible to estimate its concentration at the beginning of each cycle (3). This concentration is equal to the one that was measured at the end of the previous cycle diluted with fresh medium.

3 $$\left[Ci\right]=\left[C{i}^{{\prime} }\right]\left(\frac{Vr}{Vtot}\right)$$

Where:

[Ci’] = Concentration of *C. albicans* at the end of the previous feeding cycle (afu/mL)

Vr = Residual volume present in the proximal colon compartment (Vr = 250 mL)

Vtot = Total volume present in the proximal colon compartment including the residual volume, and the fresh feed added at the beginning of each feeding cycle (Vtot = 250 + 100 mL)

Having *C. albicans* concentrations both at the beginning and at the end of each cycle facilitated the calculation of the specific growth rate for each cycle (4).

4 $$\mu =\frac{P}{[Ci]}=\frac{\left[Cf\right]-[Ci]\,}{\left[Ci\right]\times dt}$$

Where:

μ = Specific growth rate (expressed in cycle^−1^, alternatively in hours^−1^, considering 1 cycle = 8 h)

These calculations are only estimates based on measurements and data available, hence, several limitations need to be considered, and include: (i) concentrations that did not reach the limit of quantification through qPCR were considered zero; (ii) we linearly extrapolated the concentrations between two sampling points while no information was available to confirm this linear behavior; and (iii) there was considered to be no *C. albicans* growth during the time of addition of the feed within the cycle (i.e., during the time of the dilution).

Data at genus level were processed after filtering out ASVs that were present in less than 5% of the samples at an average abundance lower than 0.01%, and after applying a total sum scaling (TSS) normalization. The gut bacteriome α-diversity was assessed by calculating Shannon and Inverse Simpson indexes using the *estimate_richness()* function from the *phyloseq* R package (v1.44.0)^74^. β-diversity was assessed using Bray–Curtis dissimilarities, computed via the *vegdist()* function from the *vegan* R package (v2.6-6.1). Principal Coordinates Analysis (PCoA) was performed using the *ordinate()* function from *phyloseq*, and Non-metric Multidimensional Scaling (NMDS) was performed using the *metaMDS()* function from *vegan*. NMDS was based on Bray–Curtis dissimilarities with a maximum of 100 iterations (trymax = 100). Sample coordinates from the ordination were combined with the corresponding metadata to visualize microbial community clustering over time and between donors or treatments. Ellipses representing 95% confidence intervals were fitted per *Candida albicans* concentration range using *stat_ellipse()* under a multivariate normality assumption. Spiders (segments) connecting individual samples to their respective group centroids were added for visualization of intra-group dispersion. All plots were generated using *ggplot2*.

*MaAsLin2* (v1.18) R package was used to identify bacterial ASVs significantly correlating with *C. albicans* concentrations^38^. To ensure robust and biologically meaningful associations, only samples collected three days after *C. albicans* inoculation were included in the analysis. This filtering step was implemented to avoid potential bias from the inoculation event itself. Indeed, within the first two days following inoculation, *C. albicans* concentrations may reflect the residual inoculum rather than active growth, especially in bioreactors where the strain fails to engraft. In such cases, high concentrations may still be detected despite the absence of microbial growth. Since non-growing microbes are typically washed out of the system within three days due to continuous dilution by feed and pancreatic juice, restricting the analysis to samples beyond this time point ensures that the observed *C. albicans* concentrations reflect actively growing populations rather than transient inoculum carryover. Furthermore, we only considered the bioreactors treated with clindamycin to ensure equal representation of each donor microbiome background, thereby standardizing their contribution to the statistical analysis. Briefly, the selected ASVs had a prevalence higher than 10%, and abundance higher than 0.01% across samples. Data was normalized using total sum scaling (normalization = “TSS”), and log-transformed (transform = log), but not standardized (standardize = FALSE), prior to analysis. They were then regressed against *C. albicans* concentrations using Linear Models (analysis_method = “LM”), with Donor as random effect to account for inter-individual variability, and day of sampling as a continuous fixed effect to account for temporal shifts. Multiple testing correction was performed using the Benjamini-Hochberg method (correction = “BH”), and significance was defined at a false discovery rate (FDR) of 0.3.

Spearman’s correlation tests of bacteriome α-diversity (Shannon’s and Simpson’s indices), bacterial concentrations, and metabolite concentrations (acetate, propionate, butyrate, or lactate) with *C. albicans* concentrations were computed using GraphPad Prism v10.1.0 (316) for Windows (GraphPad Software, San Diego, CA, USA), considering only samples collected three days post *C. albicans* inoculation as well.

*Tax4Fun2* (v1.1.5) R package was utilized to predict pathways and functional capabilities of prokaryotic communities based on 16S rRNA gene amplicon sequencing data. Correlations with *C. albicans* concentrations were measured^54^, focusing on samples collected three days post-*C. albicans* inoculation and bioreactors treated with clindamycin. *Tax4Fun2* pathway and functional predictions were performed using the default reference dataset Ref99NR, with a minimal identity similarity cutoff of 97%. Functional profiles were normalized by the number of 16S rRNA genes identified in each genome to account for differences in rRNA copy numbers. Spearman’s rank correlation coefficients were calculated using the *rcorr()* function from the *Hmisc* package (v5.1-3). The p-values were subsequently adjusted using the Holm method for multiple comparisons with the *p.adjust()* function (Stats v4.4.1). Significant correlations were visualized using the *pheatmap* package (v1.0.12).

## Supplementary information

Supplementary Information

Supplementary Data

## Data Availability

The raw Illumina sequences and corresponding metadata can be consulted under the accession number PRJNA1064676. All other raw data is available in the Supplementary Data.
