# Supplementary material for: Candida albicans colonization in the human colon correlates with a reduction in acetate- and butyrate-producing bacteria, as simulated using the M-SHIME® model
Source: NPJ Biofilms Microbiomes. 2025 Aug 26;11:176. doi: 10.1038/s41522-025-00803-w (PMC12381105; doi:10.1038/s41522-025-00803-w)
Supplement: Supplementary file 1 — Supplementary Information [file 41522_2025_803_MOESM1_ESM.pdf]

***Candida albicans* colonization in the human colon correlates with a reduction in acetate- and butyrate-producing bacteria, as simulated using the M-SHIME® model**

Benoît Marsaux [1,2,#], Warre d'Hoker [2], Frédéric Moens [1], Dries Van Elst [1], Yorick Minnebo [2], Massimo Marzorati [1,2], Tom Van de Wiele [1,2]

[1] ProDigest B.V., Ghent, Belgium
[2] CMET, Ghent University, Ghent, Belgium
[#] Correspondence: Benoit.Marsaux@Prodigest.eu

Supplementary information

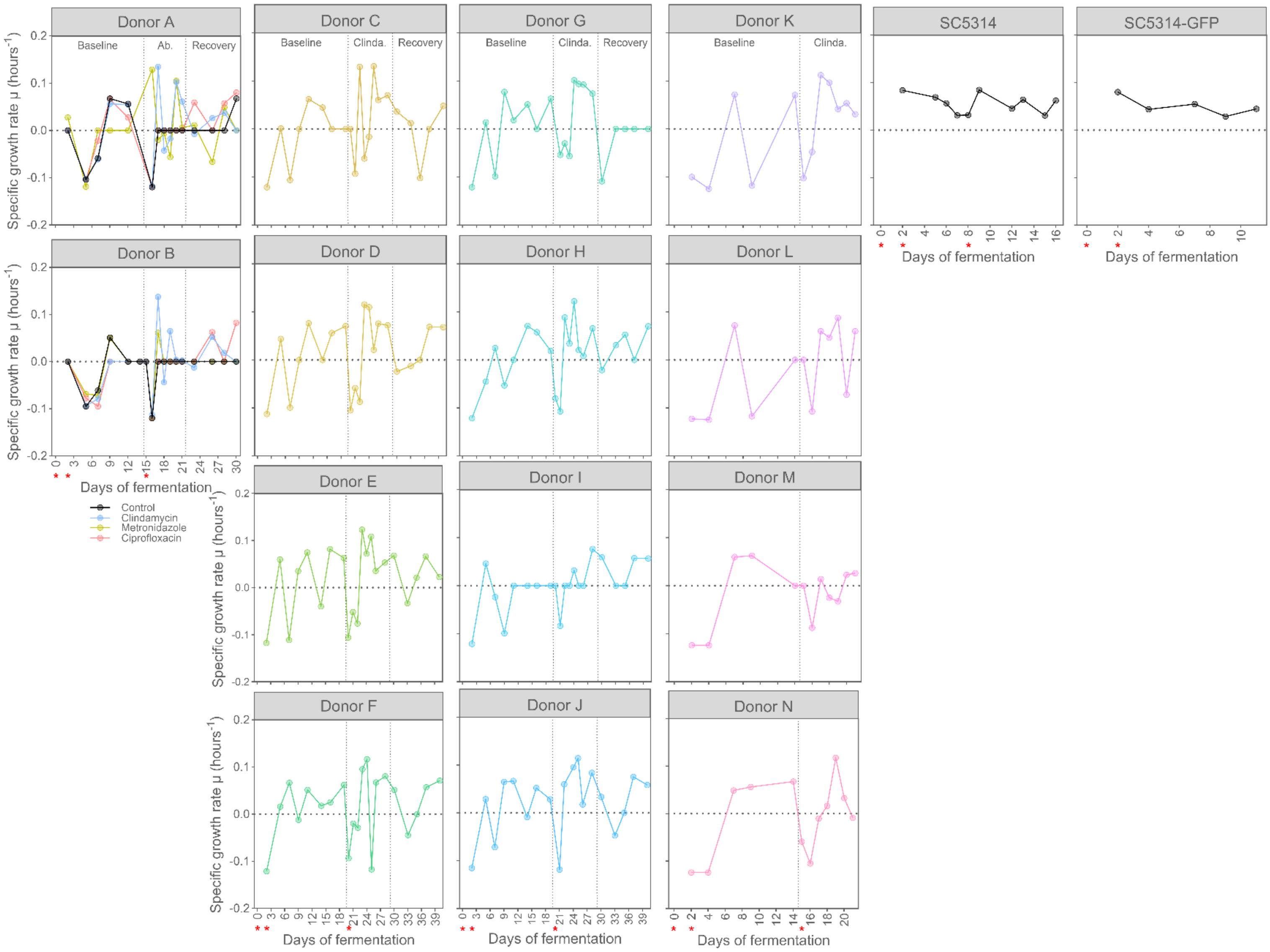

Figure S1. *Candida albicans* estimated specific growth rates at each measured time point. *Candida albicans* inoculations are indicated by a red asterisk. The antibiotic treatment period is represented by vertical dotted lines. Strain SC5314 was introduced in experiments performed with donors A to J, while SC5314-GFP was used alongside donors K to N. Ab. = antibiotic; Clinda. = clindamycin.

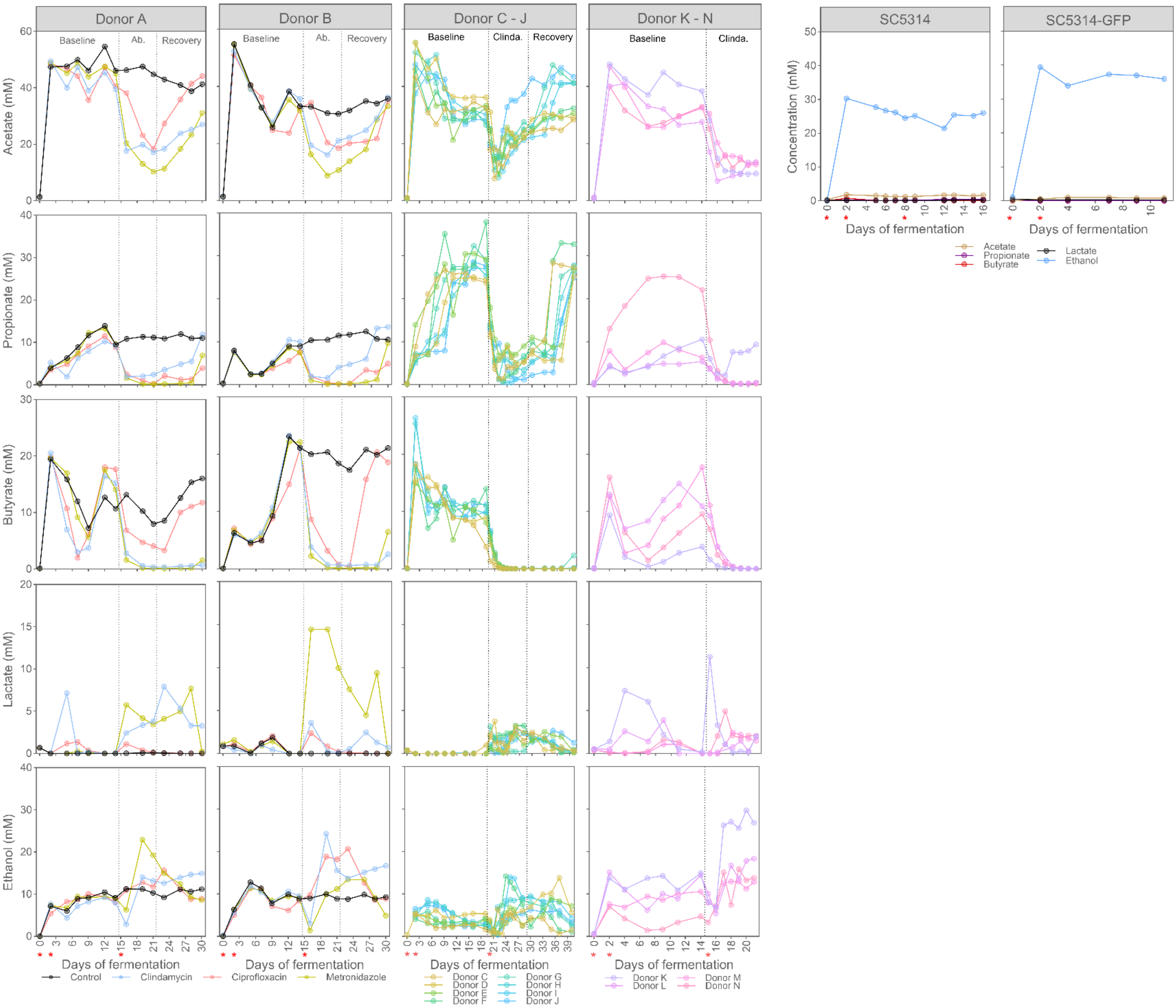

Figure S2. Acetate, propionate, butyrate, lactate, and ethanol concentration (n=1) in bioreactors inoculated with *C. albicans* only, or together with the faecal-derived microbiome from donors A to N, and grown under simulated proximal colon physiological conditions. Strain SC5314 was introduced in experiments performed with donors A to J, while SC5314-GFP was used alongside donors K to N. *Candida albicans* inoculations are indicated by a red asterisk. The antibiotic treatment period is represented by vertical dotted lines. Ab. = antibiotic; Clinda. = clindamycin.

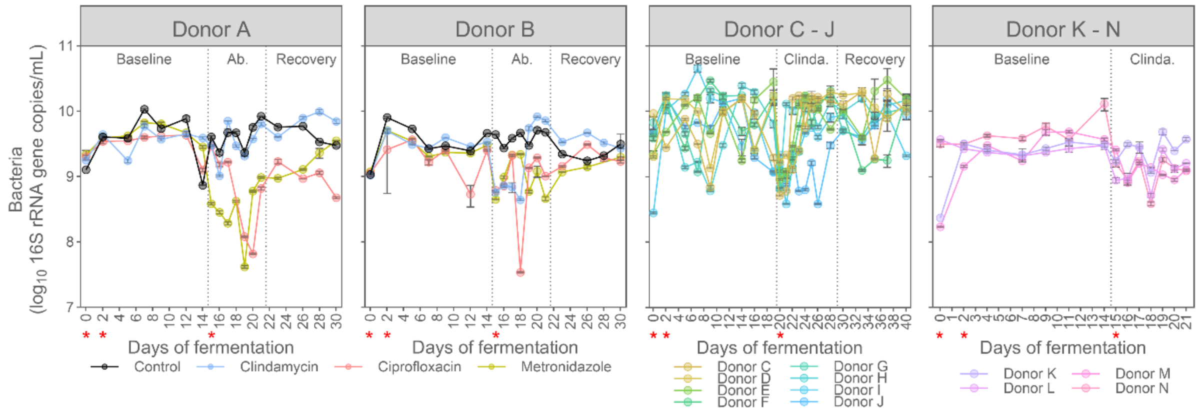

Figure S3. Total bacteria concentration measured by qPCR (n=3) in bioreactors inoculated with *C. albicans* and the faecal-derived microbiome from donors A to N, and grown under simulated proximal colon physiological conditions. Data are represented as mean ± standard deviation. Strain SC5314 was introduced in experiments performed with donors A to J; while SC5314-GFP was used alongside donors K to N. *Candida albicans* inoculations are indicated by a red asterisk. The antibiotic treatment period is represented by vertical dotted lines. Ab. = antibiotic; Clinda. = clindamycin

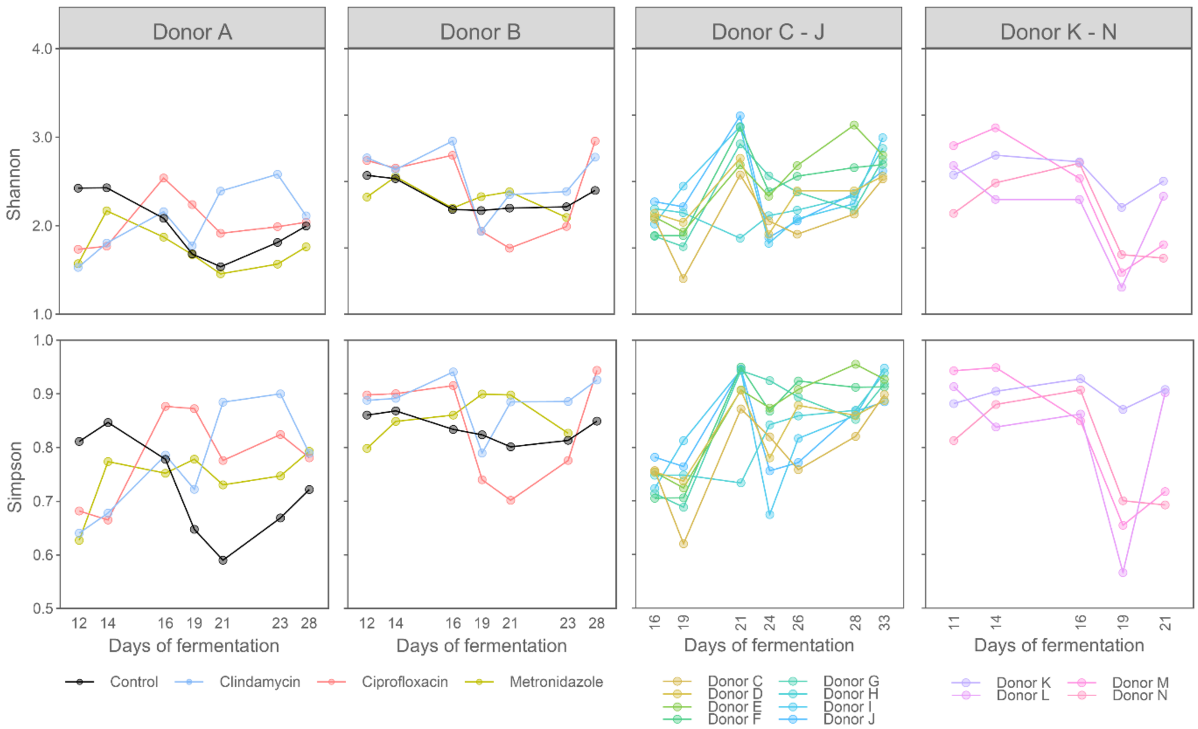

Figure S4. Shannon and Simpson bacteriome α-diversity index from samples collected throughout the different experiments inoculated with *C. albicans* and the faecal-derived microbiome from various donors. Strain SC5314 was introduced in experiments performed with donors A to J, while SC5314-GFP was used alongside donors K to N.

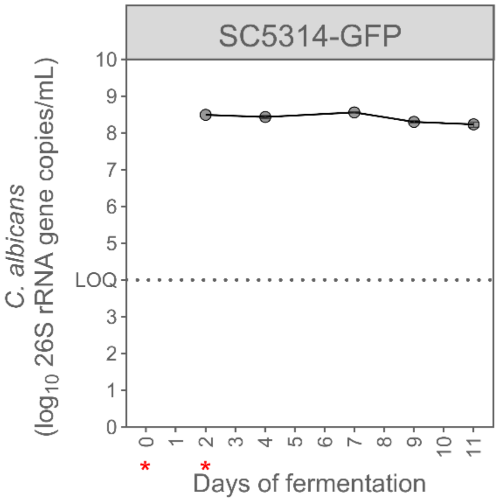

Figure S5. *Candida albicans* concentration measured by qPCR (n=3) in a microbiome-free environment under simulated proximal colon physiological conditions. Data are represented as mean ± standard deviation. A GFP reporter strain of *Candida albicans* SC5314 was inoculated at days 0, and 2 in a sterile bioreactor. *Candida albicans* inoculations are indicated by red asterisks. The limit of quantification (= LOQ) is indicated with an horizontal dotted line.

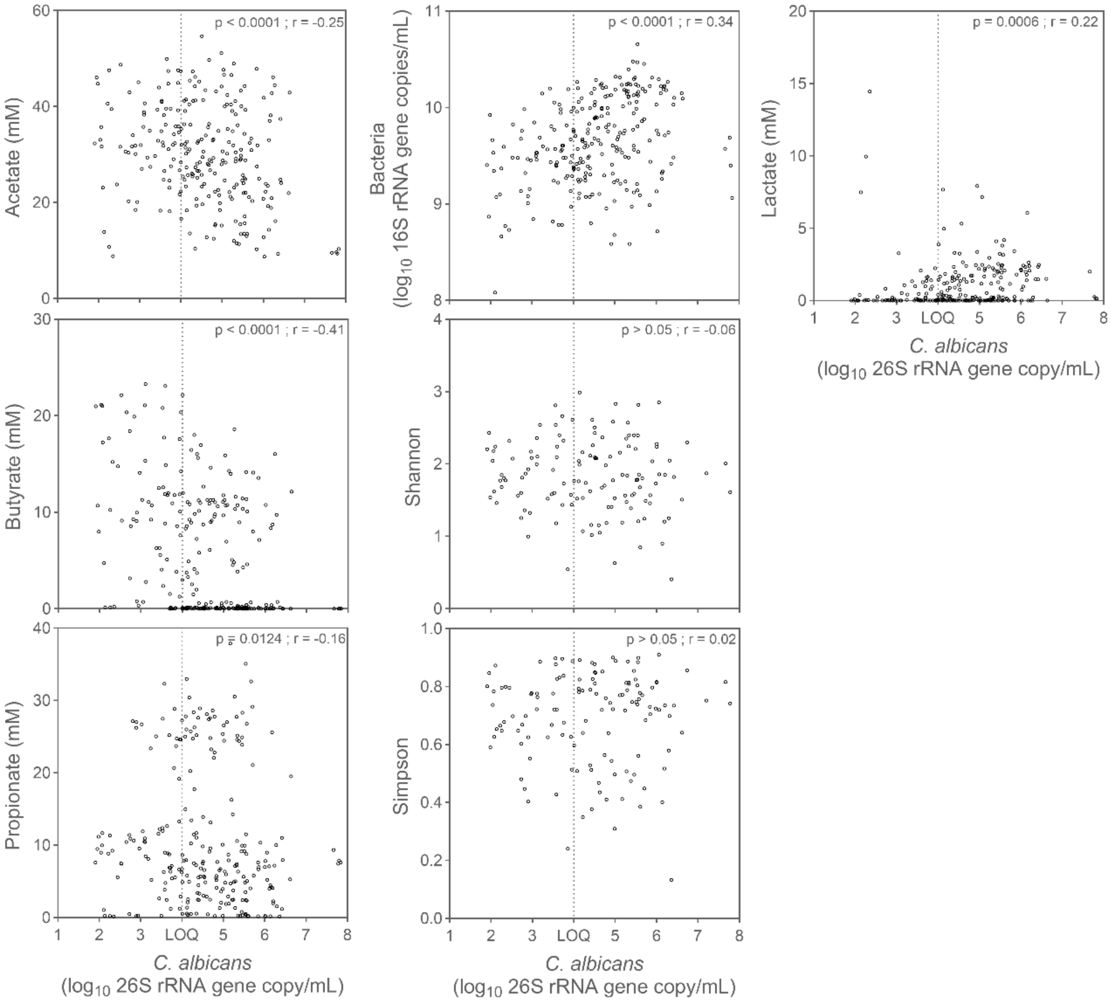

Figure S6. Dot plots of various readouts in function of *C. albicans* concentrations. The data were used to perform the Spearman's correlation tests; the obtained coefficient (r) and p-values are indicated on each plot. The limit of quantification (= LOQ) of *C. albicans* concentration is indicated by a dotted vertical line.

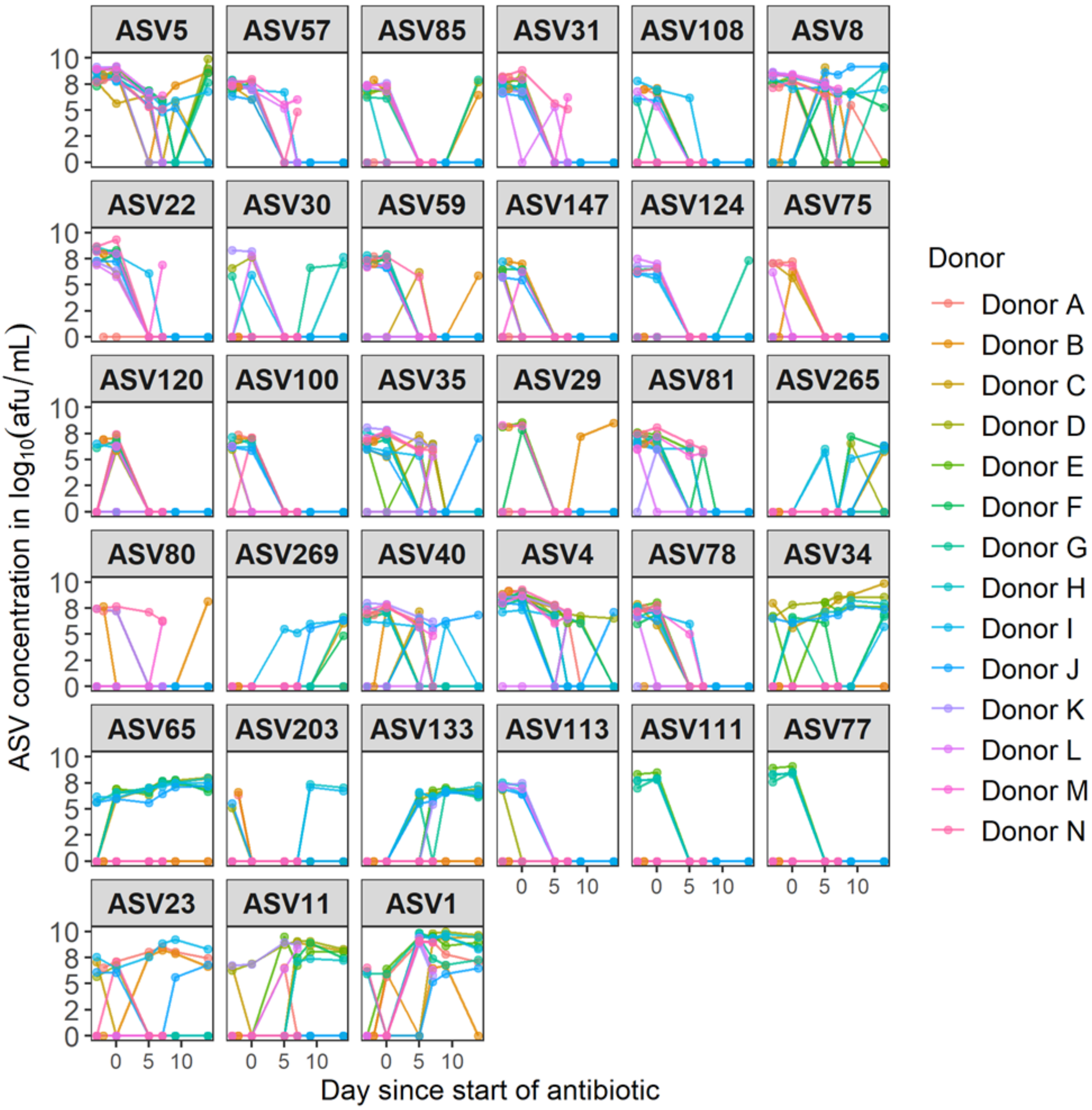

Figure S7. Concentration of the amplicon sequence variants (ASV) that were associated with *C. albicans* concentration in function of time in all the simulated colon microbiomes from donors A to N. The name of the ASVs can be found in Table 1. The relative abundance of every ASV that was significantly correlated with *C. albicans* concentrations was corrected with flow cytometry and log_{10}-transformed. Due to different experimental designs, the timeline was adjusted so that day 0 corresponds to the start of the antibiotic treatment. The antibiotic administration lasted one week for all experiments. Strain SC5314 was introduced in experiments performed with donors A to J, while SC5314-GFP was used alongside donors K to N. Facets are ordered by effect size, from most negative to most positive (top left to bottom right) from the MaAsLin2 analysis.

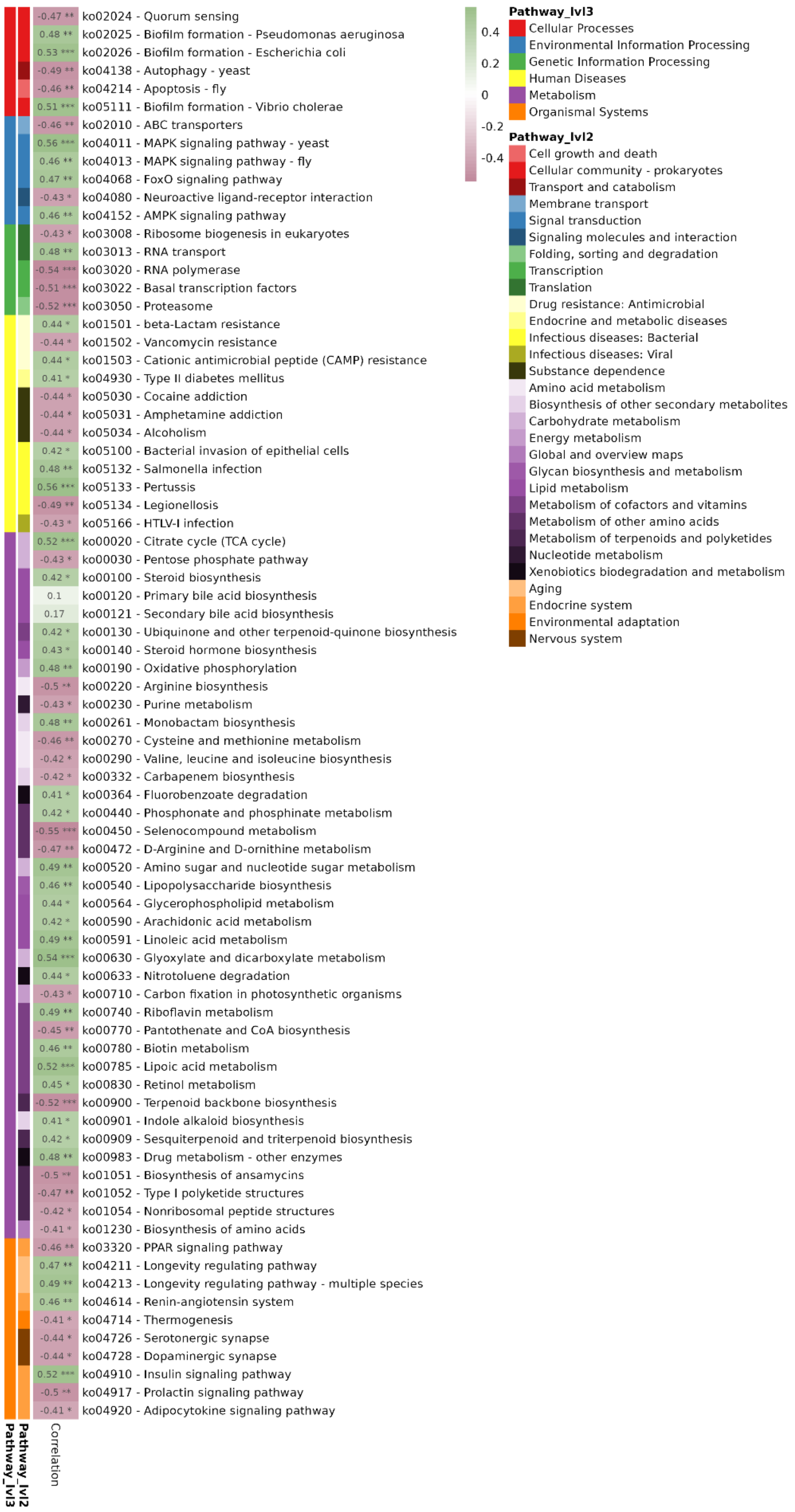

Figure S8. Heatmap of significant correlations between predicted pathways and *C. albicans* concentrations. The pathways involved in bile acid synthesis ko00120 and ko00121 are included, but not significant (p = 0.38 and 0.15, respectively). Samples were collected three days after *C. albicans* inoculation from bioreactors treated with clindamycin. Each tile displays Spearman's rank correlation coefficients (ρ) and corresponding p-values (*$p < 0.05$, **$p < 0.01$, ***$p < 0.001$, holm corrected).

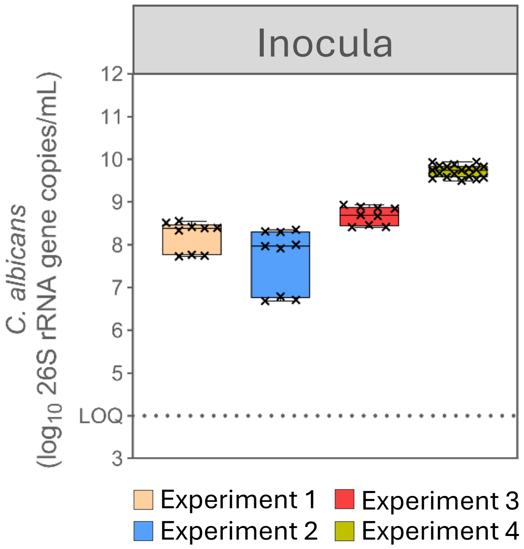

Figure S9. *Candida albicans* concentrations in the inocula used for each experiment, as measured by qPCR (n=3 at each inoculation day). Data are represented as box and whiskers plots with min-max and all points. *Candida albicans* concentrations were measured from the overnight cultures that were used to inoculate the bioreactors. Despite *C. albicans* inoculum concentrations varied across experiments, one-sample t-test comparing the observed inoculum values (excluding the outlier at day 0 from the Experiment 2) to the overall mean (8.95 ± 0.73 $\log_{10}$(26S rRNA copies/mL)) showed no statistically significant differences across experiments (p = 0.17), suggesting that the observed range is not likely to have confounded the results.
